# A new species of early-diverging Sauropodiformes from the Lower Jurassic Fengjiahe Formation of Yunnan Province, China

**DOI:** 10.1038/s41598-020-67754-4

**Published:** 2020-07-03

**Authors:** Claire Peyre de Fabrègues, Shundong Bi, Hongqing Li, Gang Li, Lei Yang, Xing Xu

**Affiliations:** 1 Centre for Vertebrate Evolutionary Biology, Institute of Palaeontology, Yunnan University, Kunming, 650091 China; 2 Department of Biology, Indiana University of Pennsylvania, Indiana, PA 15705 USA; 3 Yimen Administration of Cultural Heritage, Yimen, 651100 China; 4 Key Laboratory of Evolutionary Systematics of Vertebrates, Institute of Vertebrate Paleontology and Paleoanthropology, Chinese Academy of Sciences, Beijing, 100044 China; 5 Center for Excellence in Life and Paleoenvironment, Beijing, 100044 China

**Keywords:** Palaeontology, Taxonomy

## Abstract

Sauropodomorpha were herbivorous saurischian dinosaurs that incorporate Sauropoda and early-diverging sauropodomorphs. The oldest sauropodomorph remains are known from Late Triassic deposits, most of them Gondwanan. The Laurasian record comprises some Triassic forms, but the bulk is Jurassic in age. Among the 14 Jurassic non-sauropodan sauropodomorphs from Laurasia described in the past, 8 are from China. Here we describe a new non-sauropodan sauropodomorph, *Irisosaurus yimenensis* gen. et sp. nov., from the Early Jurassic Fengjiahe Formation of China. Nearly all of the non-sauropodan sauropodomorph genera currently known from China were first reported from the Lufeng Formation. The Fengjiahe Formation is its Southern equivalent, bringing a fauna similar to that of the Lufeng Formation to light. The new genus is defined based on an incomplete but unique maxilla, with a premaxillary ramus higher than long prior to the nasal process, a large and deep neurovascular foramen within the perinarial fossa, and a deep perinarial fossa defined by a sharp rim. Phylogenetic analysis places *Irisosaurus* at the very base of Sauropodiformes, as the sister-taxon of the Argentinean genus *Mussaurus*. This specimen adds to a growing assemblage of Chinese Jurassic non-sauropodan sauropodomorphs that offers new insight into the Laurasian evolution of this clade.

## Introduction

Sauropodomorph dinosaurs appeared during the Late Triassic. The most ancient forms have been recovered in Gondwana, although several Triassic genera were also found in Laurasia. By the Early Jurassic, the clade was most likely present on all the continents, even though specimens are still to be retrieved from Oceania^1^. At that time, one hotspot for non-sauropodan sauropodomorph diversity was Southern Asia. In this context, Southern China, and particularly the Province of Yunnan and its many Lower Jurassic strata, is an optimal location to study sauropodomorph Jurassic paleobiodiversity and the underlying evolutionary processes.

**Figure 1:**
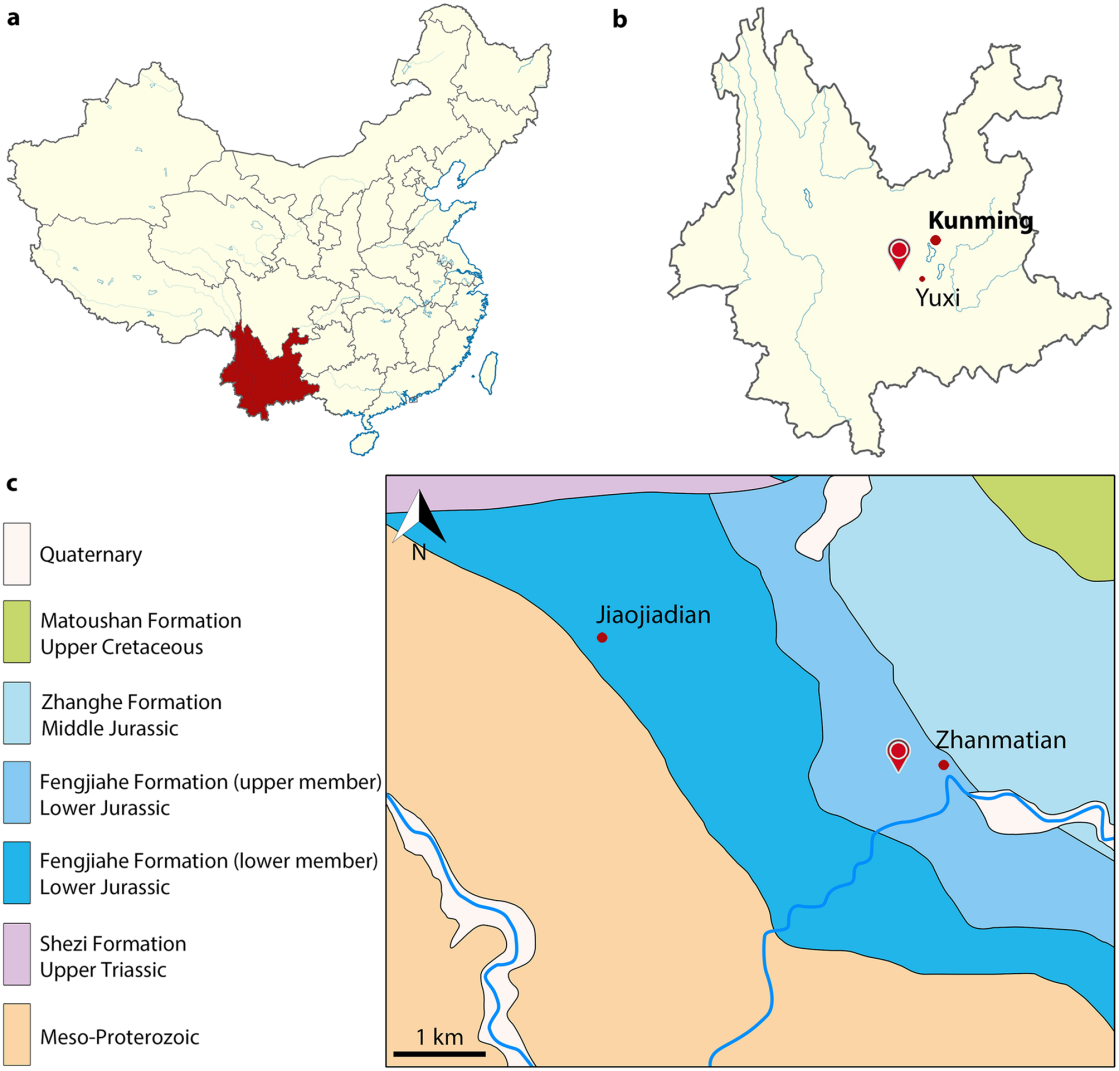
Geographic position and geology of the *Irisosaurus yimenensis* gen. et sp. nov. locality. (**a**) Map of China, showing the Yunnan Province in red; (**b**) Map of Yunnan Province, showing the location of the Province capital Kunming and Yuxi city relative to the locality position; (**c**) Geological map of the Zhanmatian area, showing the location of the locality in the Lower Jurassic Fengjiahe Formation. Maps drawn with ADOBE Illustrator 22.0 (https://www.adobe.com/products/illustrator.html).

**Figure 2:**
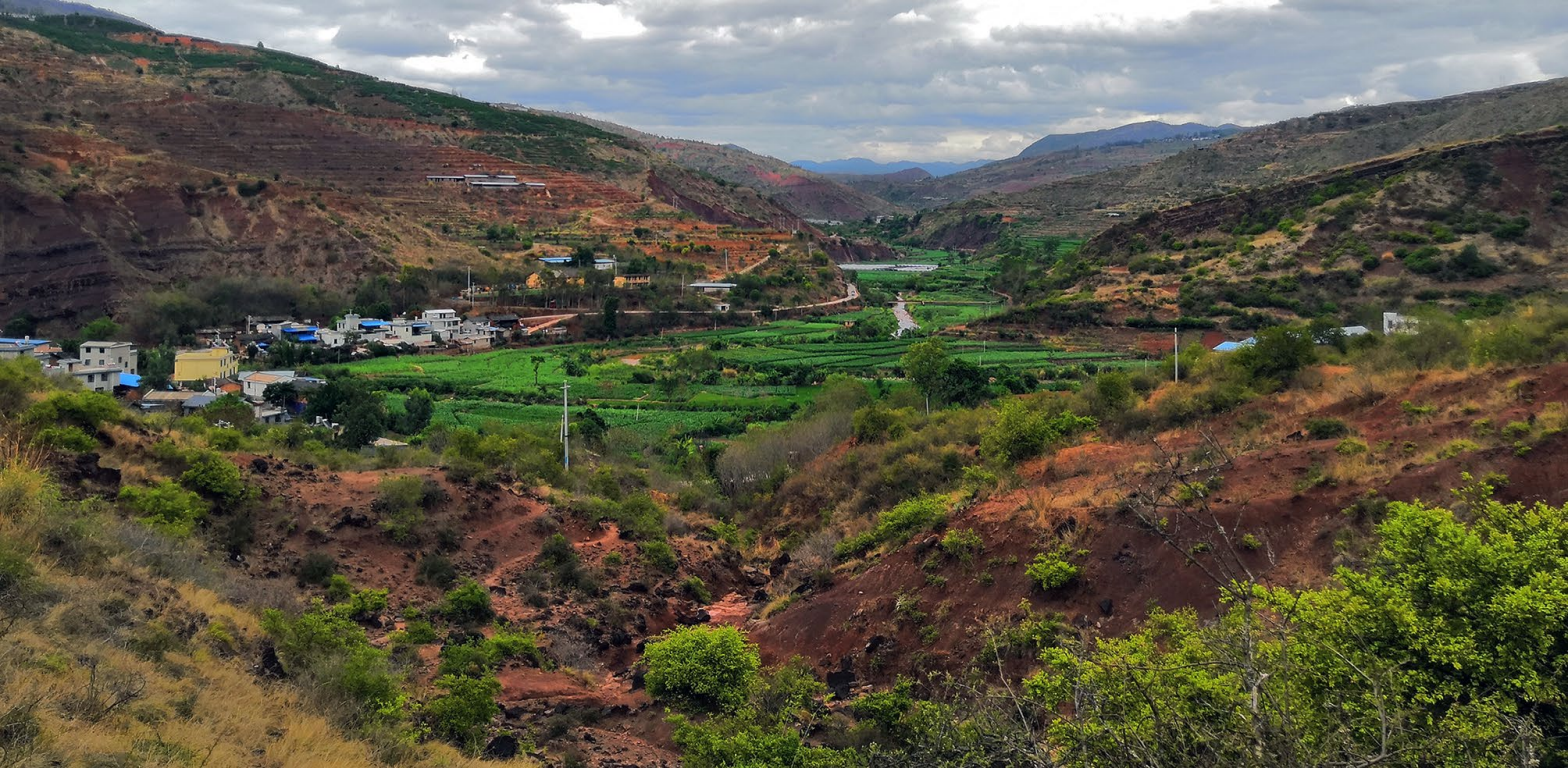
Field photograph taken near *Irisosaurus yimenensis* gen. et sp. nov. locality, showing the village of Zhanmatian on the left and the Early Jurassic reddish argillaceous siltstones surrounding it.

The abundance of remains in Yunnan has been known since the 1940s, when Young described the first sauropodomorph skeletons^2,3^. Two new species were published in the 1990s^4,5^, but it is during the last 10 years that the most important contribution to the diversity of Chinese early-diverging sauropodomorphs was made^6–9^. Nearly all the published genera (7 out of 8) were first reported from the Lufeng Formation. As a matter of fact, it is currently the richest Mesozoic unit of Yunnan Province, not only for dinosaurs, but also for several other groups including mammals, basal crocodylomorphs and spehnodontians^10^. The Fengjiahe Formation has been recognized as a lateral equivalent of the Lufeng Formation^11^ and yields a similar fauna, including the genera *Lufengosaurus* and *Yunnanosaurus*^12^. So far, only one non-sauropodan sauropodomorph genus: *Yimenosaurus*, is exclusively known from the Fengjiahe Formation^4^. The new genus reported here was discovered in the same strata and area, in Yimen County (Figs. 1, 2), but displays several main discrepancies with *Yimenosaurus* and with other taxa reported from both Fengjiahe and Lufeng formations.

## Results

### Systematic paleontology

Dinosauria Owen, 1842

Saurischia Seeley, 1887

Sauropodomorpha von Huene, 1932

Massopoda Yates, 2007

Sauropodiformes Sereno, 2007

*Irisosaurus yimenensis* gen. et sp. nov.

### Holotype

**Figure 3:**
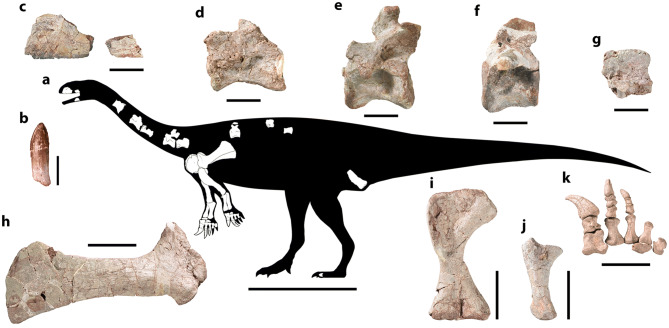
Outline of *Irisosaurus yimenensis* gen. et sp. nov. displaying the preserved material. The most informative elements are figured. (**a**) Outline; (**b**) Tooth; (**c**) Left maxilla; (**d**) Middle cervical; (**e**) Posterior cervical; (**f**) Anterior dorsal; (**g**) Middle dorsal neural spine; (**h**) Right scapula; (**i**) Right humerus; (**j**) Right ulna; (**k**) Right manus. Scale bars = 1 m (**a**);1 cm (**b**); 5 cm (**c–g**); 10 cm (**h–k**).

CVEB (Centre for Vertebrate Evolutionary Biology, Yunnan University) 21901, an associated partial skeleton (Fig. 3) including partial left maxilla (Fig. 5), partial right dentary, three isolated teeth (Fig. 5), five partial cervical vertebrae (Fig. 6; Table S1), three partial dorsal vertebrae (Fig. 7; Table S1), a dozen vertebral fragments, more than fifty dorsal rib fragments, nearly complete right scapulocoracoid (Fig. 8; Tables S2, S3) and partial left one (Fig. S1), nearly complete right humerus (Fig. 9; Table S4) and partial left one (Fig. S1), nearly complete right ulna (Fig. 9; Table S4) and partial left one (Fig. S1), complete right radius (Fig. 9; Table S4) and partial left one (Fig. S1), two partial manus with carpals (Figs. 10, S1; Table S4), two distal ends of ischia (Fig. 11; Table S5), one partial pes ungual phalanx, and several unidentifiable fragments.

### Etymology

The generic nomen refers to the famous iridescent clouds of Yunnan Province (彩云之南). The specific epithet refers to Yimen County, where the type locality is located.

### Horizon and locality

Upper member of the Fengjiahe Formation, Lower Jurassic. The specimen was collected during the summer 2018 at Jiaojiadian area B, near Zhanmatian village, Shijie Township, Yimen County, Yuxi Prefecture, Yunnan Province, China (Fig. 1). The Fengjiahe Formation is the oldest Lower Jurassic unit in the Province^13^. The fossil-bearing strata is characterized by reddish argillaceous siltstone (Fig. 2).

### Diagnosis

A small sauropodiforme distinguished from other non-sauropodan sauropodomorphs based on the following unique combination of character states (autapomorphies marked with a *): scarce and small nutrient foramina on the lateral surface of the maxilla, large and deep neurovascular foramen in the perinarial fossa*, deep perinarial fossa defined by a sharp rim*, premaxillary ramus of the maxilla higher than long prior to the nasal process*, lack of antorbital fossa (associated with the presence of a medial lamina), teeth lacking denticles (convergence with *Yunnanosaurus huangi*^3, 14^), strongly asymmetrical proximal half of metacarpal V*.

### Description

#### Skull

##### Maxilla

**Figure 4:**
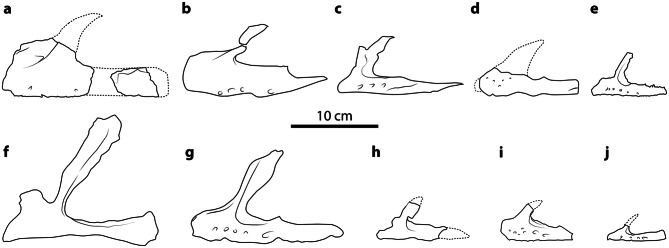
Interpretative line drawings of Asian non-sauropodan sauropodomorph maxillae in lateral view (except **f**: medial); dashed lines represent reconstructed parts and are not representative of the original shape of the bone. All drawings at the same scale. (**a**) *Irisosaurus yimenensis* gen. et sp. nov. (holotype CEVB21901); (**b**) *Jingshanosaurus xinwaensis* (holotype LFGT-ZLJ0113, modified from Zhang et al.^17^); (**c**) *Lufengosaurus huenei* (holotype IVPP V15, modified from Barrett et al.^15^); (**d**) *Pradhania gracilis* (holotype ISI R265, modified from Kutty et al.^16^); (**e**) *Xixiposaurus suni* (holotype ZLJ0108, modified from Sekiya^7^); (**f**) *Yimenosaurus youngi* (holotype YXV8701, modified from Bai et al.^4^); (**g**) *Yizhousaurus sunae* (holotype LFGT-ZLJ0033, modified from Zhang et al.^9^); (**h**) *Yunnanosaurus huangi* (holotype NGMJ 004546, modified from Barrett et al.^14^); (**i**) *Yunnanosaurus robustus* (holotype IVPP V93, modified from Young^26^); (**j**) *Yunnanosaurus robustus* (juvenile; ZMNH-M8739, modified from Sekiya et al.^18^).

**Figure 5:**
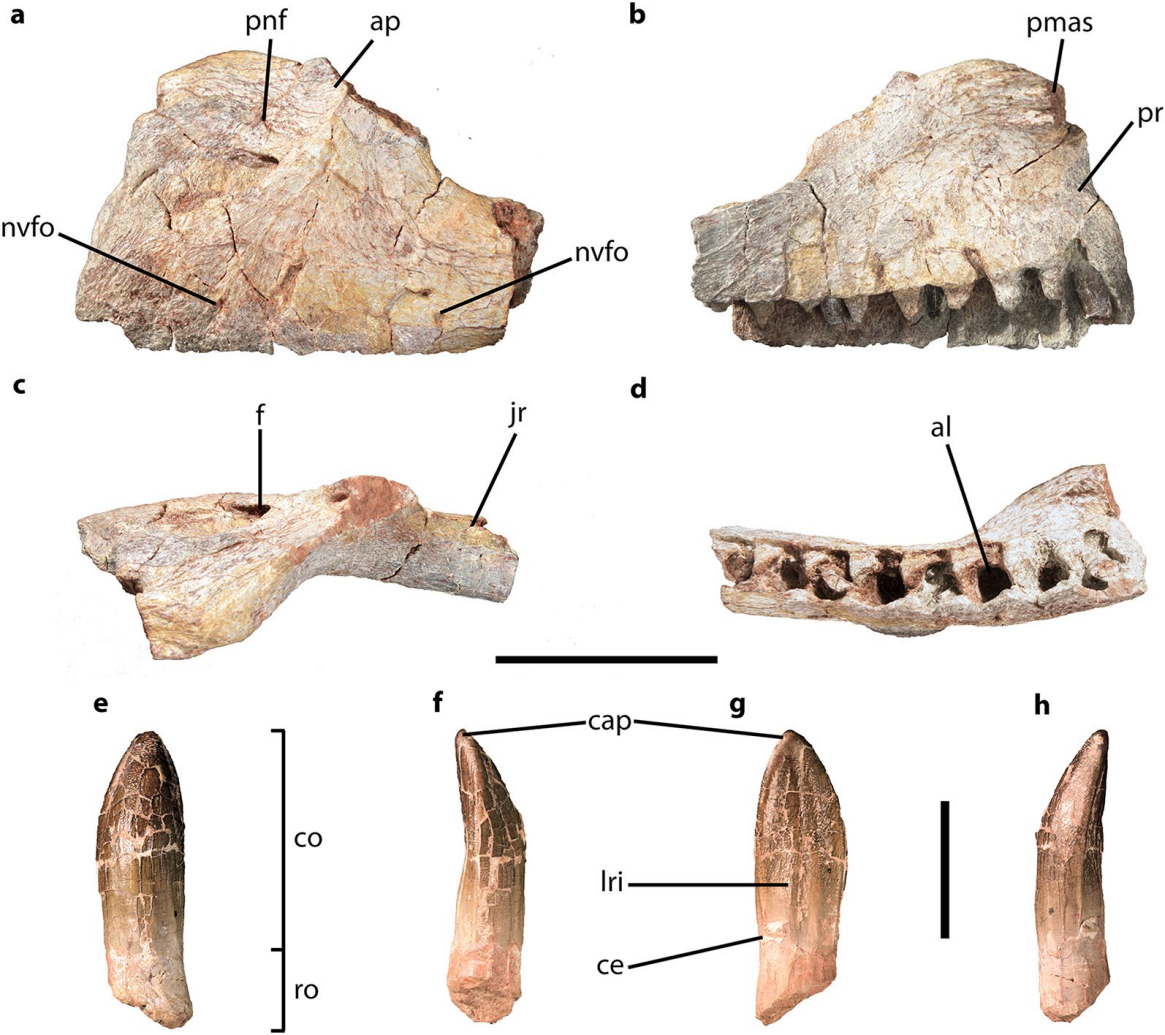
*Irisosaurus yimenensis* left maxilla (**a–d**) and isolated tooth (**e–h**). (**a**) Lateral view; (**b**) Medial view; (**c**) Dorsal view; (**d**) Ventral view; (**e**) Labial view; (**f**) Mesial or distal view; (**g**) Lingual view; (**h**) Mesial or distal view. Abbreviations: al, alveoli; ap, ascending process; cap, crown apex; ce, cervix; co, crown; f, foramen; jr, jugal ramus; lri, longitudinal ridge; nvfo, neurovascular foramen; pmas, premaxillary articular surface; pnf, perinarial fossa; pr, premaxillary ramus; ro, root. Scale bars = 5 cm (**a–d**); 1 cm (**e–h**).

The left maxilla consists of the anterior part of the main tooth-bearing body and a fragment of posterior ramus (Figs. 4, 5). The lateral surface of the maxilla does not bear a lateral maxillary ridge, like in *Lufengosaurus*^15^. Only two very small neurovascular foramina are visible on the lateral surface of the premaxillary ramus, and none is observed on the posterior ramus. This is a surprising feature, given that in most Jurassic non-sauropodan sauropodomorphs the maxilla has numerous (4–8) large neurovascular foramina, most of which are located on the anterior portion of the maxilla (Fig. 4). The closest condition to that of *Irisosaurus* has been described in *Yunnanosaurus huangi*^14^, in which the complete absence of foramina is considered an autopomorphy.

The portion of the premaxillary ramus prior to the nasal process is short, as in *Lufengosaurus*^15^ and *Pradhania*^16^. It is also markedly expanded dorsoventrally relative to its anteroposterior development. In most other genera, such as *Jingshanosaurus*^5,17^, *Lufengosaurus*^15^, *Xixiposaurus*^7^, *Yimenosaurus*^4^ and *Yizhousaurus*^9^, the premaxillary ramus is longer than high. On the anterodorsal portion of the premaxillary ramus, the base of the premaxillary articular surface is strongly deflected medially. The premaxillary ramus of the maxilla bears an elongate curved ridge delimiting the perinarial fossa. Only two other Asian taxa (*Jingshanosaurus*^5,17^ and *Xixiposaurus*^7^) display similarly elongate ridges. The extension of the perinarial fossa on the maxilla is observed in several Chinese forms in which the skull is preserved: *Jingshanosaurus*^5,17^, *Lufengosaurus*^15^, *Xixiposaurus*^7^, *Yizhousaurus*^9^ and *Yunnanosaurus* (pers. obs., ZLJ0110). Along the ridge, the perinarial fossa incorporates a large and deep neurovascular foramen opening anteriorly. This feature is not present in any other early-diverging sauropodomorph for which appropriate material is known. We therefore consider it an autapomorphy of *Irisosaurus*.

Posterior to the nasal process, the maxilla bears a marked concavity corresponding to the anteroventral corner of the antorbital fenestra. Surprisingly, and conversely to what is observed in most non-sauropodan sauropodomorphs, no antorbital fossa (associated with the presence of a medial lamina) is visible. To our knowledge, this feature is not observed in any other genus, except maybe *Pradhania*, for which it is not specified in the description^16^.

Along the alveolar margin, eight alveoli are preserved on the anterior of the maxilla, and four on the posterior ramus fragment. Five alveoli show replacement teeth preserved in situ.

##### Dentary

The dentary is only represented by a small fragment from the right side. The dorsal margin of the preserved part diverges from the ventral margin, so that the posterior dorsoventral depth of the fragment is approximately twice its anterior dorsoventral depth. The fragment is identified as the posteriormost part of the dentary, close to the articulation with the surangular and angular.

##### Dentition

Besides maxillary teeth, three isolated teeth were recovered (Fig. 5). They are straight folidont, labiolingually compressed, with a reniform cross-section^19^. The crown is spatulate, with a strongly convex labial surface and a smoothly concave lingual surface, closer to what is observed in *Lamplughsaura*^16^, *Yunnanosaurus*^3,14^, *Leonerasaurus*^20^, and *Pulanesaura*^21^, than in other non-sauropodan sauropodomorphs. The mesiodistally widest point of the crown is not located at the base of the crown, but at mid-height. This condition is also observed in *Jingshanosaurus*^5,17^, *Lamplughsaura*^16^, *Leonerasaurus*^20^ and *Yunnanosaurus*^3,14^. The Slenderness Index (‘SI’: length of the tooth crown divided by its maximum mesiodistal width^22^) of *Irisosaurus* is 2.36, that is higher than *Lufengosaurus* (1.70) and *Yunnanosaurus* (1.95), but lower than *Yimenosaurus* (2.43)^23^.

The carinae do not bear denticles, conversely to the condition of most non-sauropodan sauropodomorphs except *Yunnanosaurus huangi*^14^.

The tooth enamel is smooth at the base of the crown and finely wrinkled more apically. Finely wrinkled tooth enamel has also been described in one specimen of *Jingshanosaurus*^17^ and in *Lamplughsaura*^16^. On the labial surface, several longitudinal small blunt bulges are visible at the base of the crown. On the lingual surface, the best-preserved tooth bears two central and apicobasally oriented ridges, separated by a flute.

A very slight constriction is visible at the base of the crown, at the level of cervix. At the fracture point, the root has an oval cross-section with a large pulp cavity.

#### Axial skeleton

##### Cervical vertebrae

**Figure 6:**
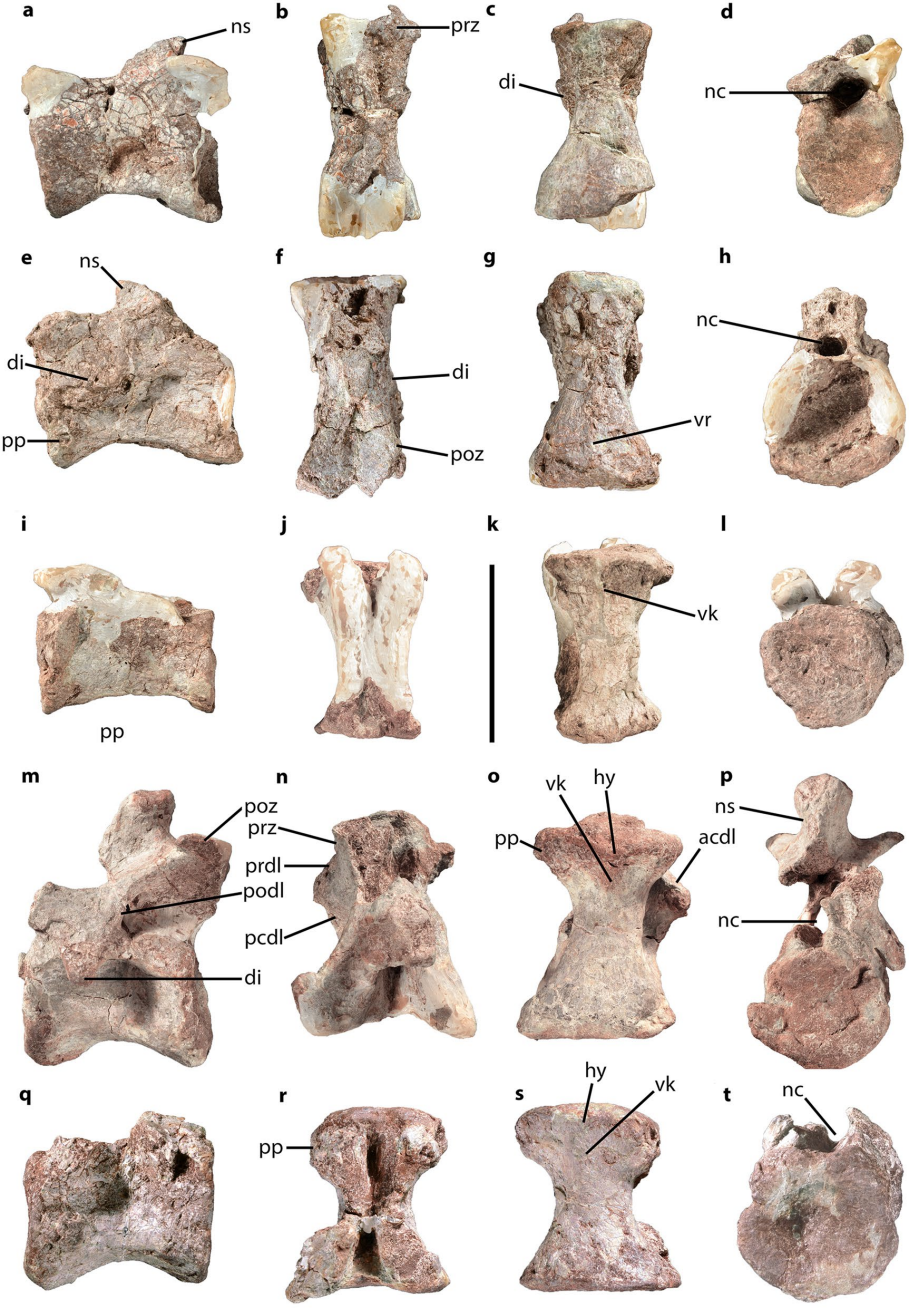
*Irisosaurus yimenensis* cervical vertebrae in, from left to right, left lateral, dorsal, ventral and anterior views. (**a–d**) Anterior cervical (C3-C5?); (**e–h**) Middle cervical (C5-C7?); (**i–l**) Middle cervical (C6-C8?) centrum; (**m–p**) Posterior cervical (C9?); (**q–t**) Posterior cervical (C10?) centrum. Abbreviations: acdl, anterior centrodiapophyseal lamina; di, diapophysis; hy, hypapophysis; nc, neural canal; ns, neural spine; pcdl, posterior centrodiapophyseal lamina; podl, postzygodiapophyseal lamina; poz, postzygapophysis; pp, parapophysis; prdl, prezygodiapophyseal lamina; prz, prezygapophysis; vk, ventral keel; vr, ventral ridge. Scale bar = 10 cm.

Five vertebrae, largely complete, were identified as cervical vertebrae (Fig. 6; Table S1). They are elongated, with no marked pneumatization visible on the centra and neural arches. They are subequal in length, with a length over height ratio of 1.9 for the longest cervical centrum. This ratio is similar to that of the longest cervical vertebra of *Lamplughsaura*^16^ and *Yizhousaurus*^9^ (2.0), and close to that calculated for *Lufengosaurus*^2,24^, *Xingxiulong*^8^, *Yunnanosaurus huangi*^3^ and *Yunnanosaurus youngi*^25^ (2.4). Otherwise, most non-sauropodan sauropodomorphs have a length over height ratio between 3 and 4.5. Cervical centra have subcircular articular surfaces and all present a ventral median constriction. The most anterior middle cervical seems to bear an incipient ventral ridge throughout its length, while the more posterior middle cervical centrum has a ventral keel on its anterior half. Both posterior cervical vertebrae exhibit a small hypapophysis merged with an anterior ventral ridge.

The parapophyses are low and located in the anteroventral corner of the centrum on middle cervical vertebrae. On posterior cervical vertebrae, parapophyses are large tubercles overlapping the anterior margin of the centrum.

The diapophyses are incipient, non-projecting, subtriangular processes on the anterior cervical vertebra, like in most non-sauropodan sauropodomorphs. On the middle cervical vertebra they rise at the level of the neurocentral suture, in the anterior half of the vertebra. On one posterior cervical vertebra, the diapophysis is more developed and located more posterodorsally. It is subtriangular in shape, and on either side of it two blunt laminae can be identified: the anterior centrodiapophyseal lamina (acdl) and the posterior centrodiapophyseal lamina (pcdl).

On one posterior cervical vertebra, a prezygodiapophyseal lamina (prdl) is visible on the neural arch, although prezygapophyses were not preserved. Posteriorly, the postzygapophysis is projecting laterodorsally and has a flat articular surface. On the lateral surface of the neural arch, a marked postzygodiapophyseal lamina (podl) is visible. In posterior aspect, an intrapostzygapophyseal lamina (tpol) and a spinopostzygapophyseal lamina (spol) are observed. The postzygapophysis does not bear any epipophysis on its dorsal surface, conversely to that of *Yizhousaurus*^9^ and *Yunnanosaurus youngi*^25^.

The neural arch below the neural spine is low on anterior and middle cervical vertebrae and high on posterior cervical vertebrae. The neural spine base goes from transversely compressed on the middle cervical vertebra to shorter and transversely thick on the posterior cervical vertebra.

##### Dorsal vertebrae

**Figure 7:**
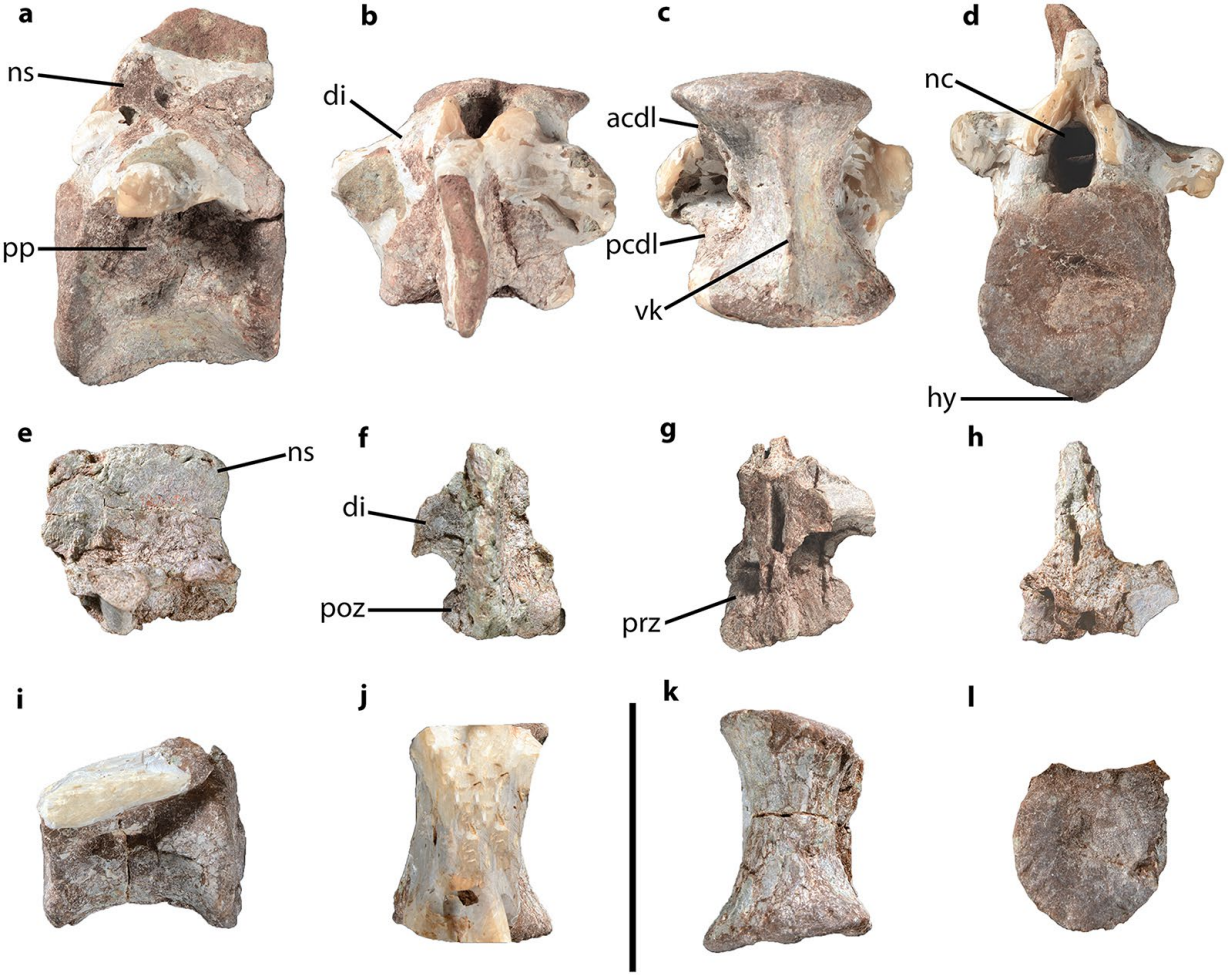
*Irisosaurus yimenensis* dorsal vertebrae in, from left to right: left lateral, dorsal, ventral and anterior views. (**a–d**) Anterior dorsal (D2-D6?); (**e–h**) Middle dorsal (D5-D9?) neural spine; (**i–l**) Middle dorsal (D5-D9?) centrum. Abbreviations: acdl, anterior centrodiapophyseal lamina; di, diapophysis; hy, hypapophysis; nc, neural canal; ns, neural spine; pcdl, posterocentrodiapophyseal lamina; poz, postzygapophysis; pp, parapophysis; prz, prezygapophysis; vk, ventral keel. Scale bar = 10 cm.

Three partial vertebrae were identified as dorsal vertebrae (Fig. 7; Table S1). The centra are higher and shorter than in the cervical vertebrae, with subcircular articular surfaces and, in ventral aspect, a marked median constriction. The anterior centrum ventral surface exhibits a hypapophysis and a marked ventral keel on its entire length. Ventral keels have also been described on anterior dorsal vertebrae of *Lufengosaurus huenei*^2^, *Xingxiulong*^8^, *Yizhousaurus*^9^ and *Yunnanosaurus*^3,25,26^.

Parapophyses of the anterior dorsal vertebra are oval-shaped and low. They are located in the dorsomedian area of the centrum, where they overlap the neurocentral suture.

On the anterior dorsal vertebra, the diapophyses base is bordered posteroventrally by a posterior centrodiapophyseal lamina (pcdl), and by an anterior centrodiapophyseal lamina (acdl) anteroventrally.

The isolated dorsal neural spine preserves both postzygapophyses, one being still articulated with the corresponding prezygapophysis. Articular surfaces are flat, circular and oriented strictly dorsally and ventrally for the prezygapophysis and postzygapophyses, respectively. Above postzygapophyses, small spinopostzygapophyseal laminae (spol) are visible on the posterior aspect. The dorsal surface of postzygapophyses bears no epipophysis.

The neural arch to vertebra height ratio of the anterior dorsal vertebra is low in comparison with the posterior cervical vertebra. The dorsal neural spine are much longer than wide, and transversely compressed.

#### Pectoral girdle

**Figure 8:**
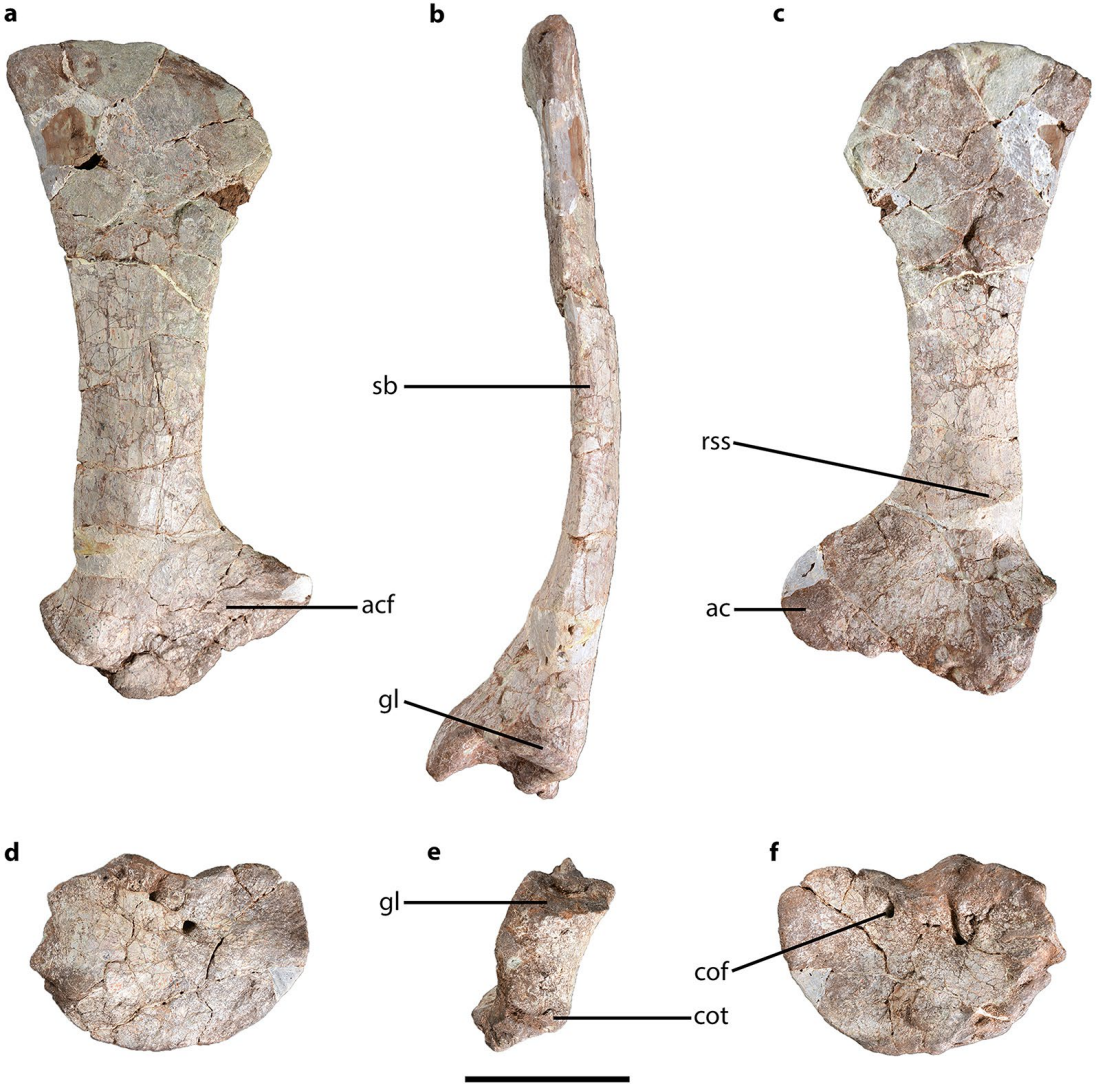
*Irisosaurus yimenensis* right scapula and coracoid in, from left to right: lateral, posterior and medial views. (**a–c**) Scapula; (**d–f**) Coracoid. Abbreviations: ac, acromion; acf, acromial fossa; cof, coracoid foramen; cot, coracoid tubercle; gl, glenoid; rss, ridge surrounding the attachment area of the muscle *serratus superficialis*; sb, scapular blade. Scale bar = 10 cm.

Both scapulae and coracoids were preserved. The right scapula and coracoid were separated during the preparation of the specimen (Fig. 8; Tables S2, S3). The left scapulocoracoid has several damaged parts (Fig. S1; Tables S2, S3).

The scapula is elongate and slender, like in *Lufengosaurus*^2^, *Xixiposaurus*^7^ and *Yunnanosaurus huangi*^3^. The proximal and distal ends are anteroposteriorly extended with respect to the scapular blade. The posterodorsal corner of the scapula most likely projected behind the posteroventral corner of the scapula, like in *Jingshanosaurus*^5^, *Yizhousaurus*^9^ or *Yunnanosaurus huangi*^3^.

The scapular blade (above the glenoid, without the distal end) represents circa 50% of the total length of the bone. In lateral view, the anterior and posterior margins of the scapular blade are subparallel on most of their length. This condition is also observed in several Chinese taxa, such as *Jingshanosaurus*^5^, *Lufengosaurus*^2,24^, *Yizhousaurus*^9^ or *Yunnanosaurus huangi*^3^. In transverse section, the blade has an oval shape. The lateral and medial surfaces of the blade are smooth, but the medial surface bears a blunt posteroventral ridge.

The proximal end of the scapula shows almost the same anteroposterior extension as its distal end. In some taxa, like *Lufengosaurus huenei*^2^, the proximal end is clearly more extended. The shallow acromion fossa extends on most of the length of the proximal end. The acromion does not project much anteriorly and its minimal height, measured on the anterior margin, represents 18% of the complete scapula. The dorsal border of the acromion is oblique, and without visible angle, as in *Jingshanosaurus*^5^, *Yizhousaurus*^9^ or *Yunnanosaurus huangi*^3^. The dorsal margin of the acromion is positioned at an angle of 120° to the main (dorsoventral) axis of the scapula. Opposite to the acromion, the sharp posterodorsal corner of the glenoid projects posteriorly. A similar degree of projection is observed in *Lufengosaurus huenei*^2^, *Yizhousaurus*^9^ and *Yunnanosaurus huangi*^3^. In contrast, the projection is not as prominent in *Jingshanosaurus*^5^ and *Lufengosaurus magnus*^24^.

The coracoid is a robust and oval-shaped bone, with a long-axis oriented anteroposteriorly. The coracoid height over length ratio equals 73%, as in *Yizhousaurus*^9^. In comparison, the ratio in *Jingshanosaurus*^5^ and *Lufengosaurus*^2,24^ is approximately 60%. The transversely thinnest part of the coracoid is on its anterior part. Conversely, the thickest area is the glenoid cavity. The lateral surface of the coracoid is slightly convex and bears a laterally projecting, rounded, tubercle. The posterior margin of the bone bears a posterodorsally oriented glenoid cavity. When in articulation, the angle between the coracoid component of the glenoid and the scapular component is approximately 100°.

#### Forelimb

##### Humerus

**Figure 9:**
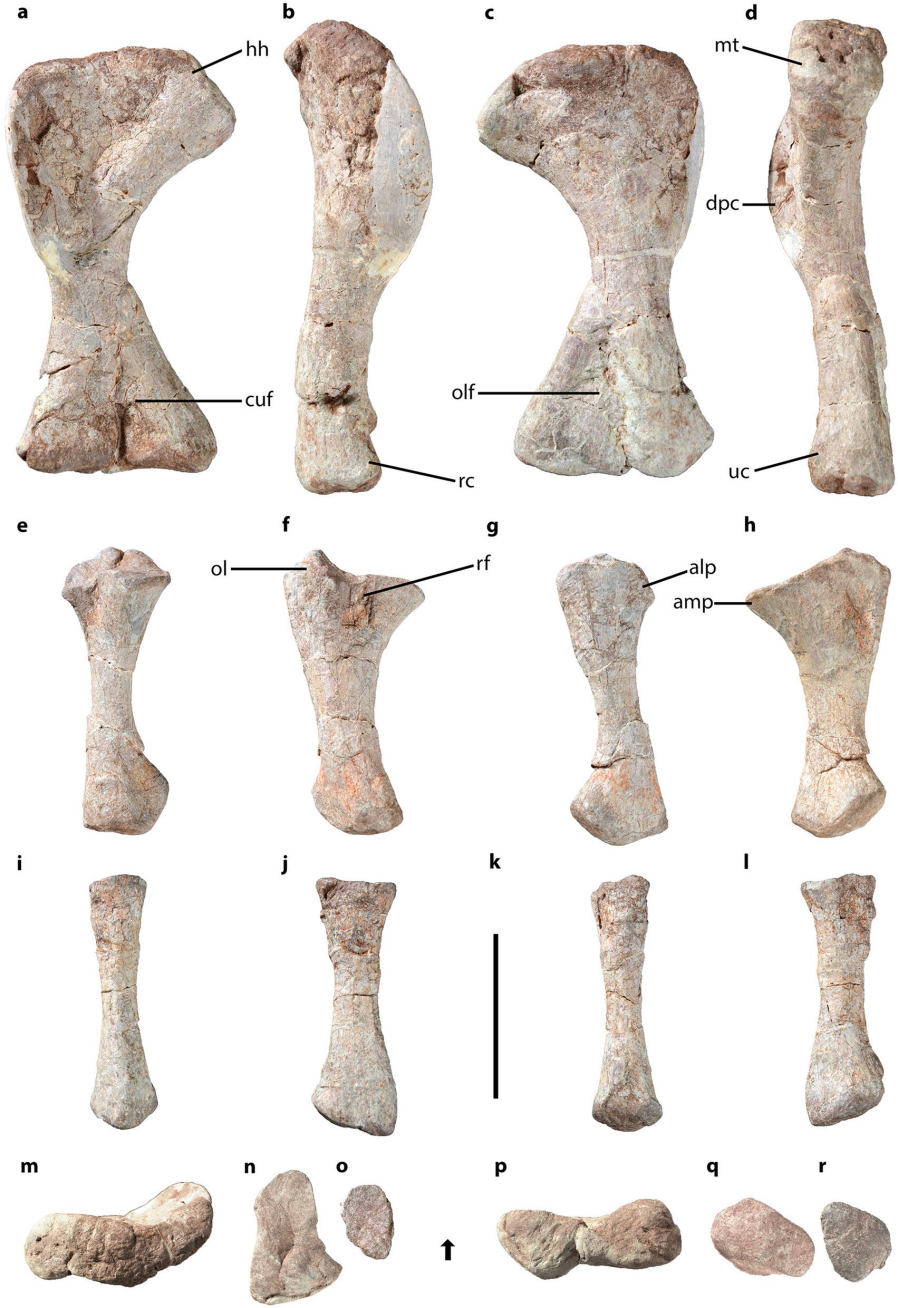
*Irisosaurus yimenensis* right humerus, ulna and radius in, from left to right: anterior, lateral, posterior and medial views. Proximal and distal views on the last line. (**a–d**,**m**,**p**) Humerus; (**e–h**,**n**,**q**) Ulna; (**i–l**,**o**,**r**) Radius. Abbreviations: alp, anterolateral process; amp, anteromedial process; cuf, cuboid fossa; dpc, deltopectoral crest; hh, humerus head; mt, medial tuberosity; ol, olecranon; olf, olecranon fossa; rc, radial condyle; rf, radial fossa; uc, ulnar condyle. The arrow indicates the anterior surface of bones for (**m**–**r**). Scale bar = 10 cm.

Both humeri were recovered; the right humerus is nearly complete, the left one incomplete (Figs. 9, S1; Table S4). The humerus is hourglass-shaped and slender. The humeral head is rounded and bears a distinct bulge. The deltopectoral crest is incomplete, and probably had a development similar to that seen in *Yizhousaurus*^9^ or *Yunnanosaurus*^3,26^. However, in *Yizhousaurus*^9^, the deltopectoral crest arises quite distally relative to the proximal margin of the bone, while in *Irisosaurus*, *Xixiposaurus*^7^ and *Yunnanosaurus*^3,26^ it arises closer to the proximal margin of the bone. The deltopectoral length is 50% of the total humerus.

The humerus diaphysis is short relatively to the total length of the bone, therefore its medial and lateral borders are strongly concave in anterior view, as in *Jingshanosaurus*^5^, *Yizhousaurus*^9^ and *Yunnanosaurus*^3,26^. Conversely, in *Lamplughsaura*^16^ and *Xixiposaurus*^7^, the diaphysis has less marked medial and lateral concavities. In lateral view, the anterior and posterior borders of the diaphysis are subparallel. In cross-section, the diaphysis is oval, its transverse width being superior to its anteroposterior thickness. In some genera, such as *Lufengosaurus*^2,24^ or *Xixiposaurus*^7^, the section is circular.

The transverse distal extension of the humerus equals 85% of the proximal extension. The anterior surface of the distal humerus bears the cuboid fossa, and the posterior surface bears a shallow olecranon fossa. The condyles are visible, but poorly developed, as is often the case in non-sauropodan sauropodomorphs. In distal view, the condyles are subequal in size. The radial (medial) condyle is circular, while the ulnar (lateral) condyle is more oval-shaped, with an oblique long-axis.

##### Ulna

Both ulnae were recovered, but the right ulna is the most complete and best preserved (Figs. 9, S1; Table S4).

The ulna to humerus length ratio is 68%. Both ends of the ulna are extended with respect to the diaphysis, and the main axis of the bone presents a rotation of approximately 40°. The proximal articular surface of the ulna has a subtriangular outline, with an oval anteromedial process longer than the anterolateral process. Between both processes is the shallow radial fossa. Another concavity, as deep as the radial fossa, is visible on the medial border. Posteriorly, the articular surface of the ulna bears the olecranon. It is a blunt projection showing the same degree of development as in *Lufengosaurus*^2,24^, *Yizhousaurus*^9^ or *Yunnanosaurus*^3,26^.

The diaphysis has a circular cross-section. Relative to both ends, it seems stockier than in *Lufengosaurus huenei*^2^, but more gracile than in *Jingshanosaurus*^5^. In lateral and medial views, both anterior and posterior margins of the diaphysis are concave, while in *Yizhousaurus*^9^ the posterior margin is rather straight.

In medial view, the posterodistal corner of the ulna is projecting posterodorsally, and is located more dorsally than the anterodistal corner. The distal articular surface is subrectangular with a distal convexity.

##### Radius

The right radius is completely preserved, whereas both ends of the left radius are damaged (Figs. 9, S1; Table S4). The radius represents 60% of the humerus length. It is a straight and slender bone, with both ends more extended than the diaphysis. Its proximal end is oval-shaped, with a flat proximal articular surface.

The diaphysis is straight, with concave margins in lateral view. It is elliptical in cross-section.

The distal end of the radius is more extended than the proximal end, transversely and anteroposteriorly. The posterodistal corner of the radius projects further than the anterodistal corner. The distal articular surface is flat and subtriangular in distal view.

##### Manus

**Figure 10:**
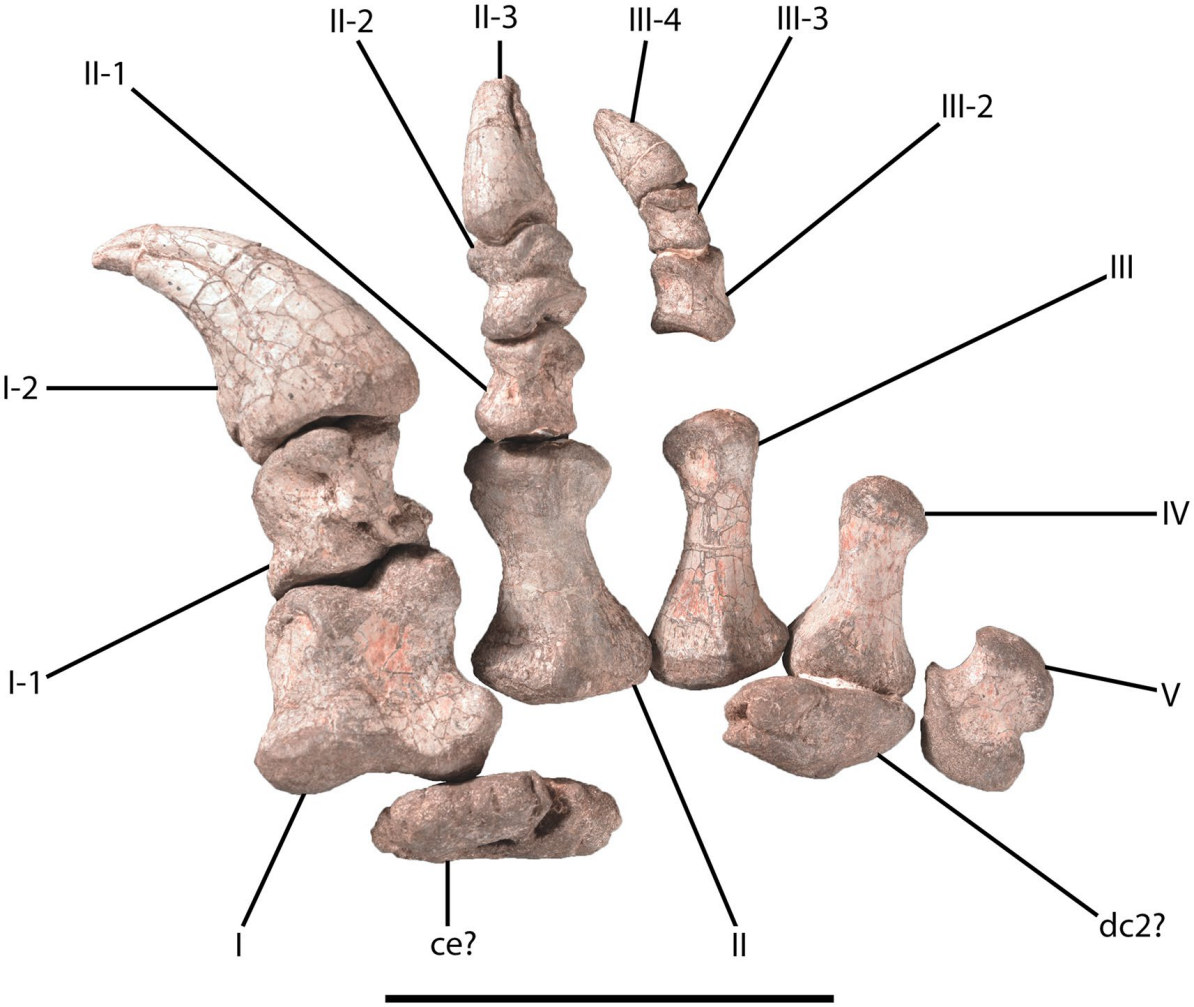
*Irisosaurus yimenensis* right manus. Abbreviations: ce, centrale; dc2, distal carpal 2; I-V, metacarpals I to V; X-x, digit X phalanx x. Scale bar = 10 cm.

Both hands were preserved, but the right hand is the most complete (Figs. 10, S1; Table S4).

The carpus includes two ossified carpals. Based on the interpretation made by Läng and Goussard^27^ (Fig. 5B) and on a compilation of fossil data, we tentatively identify the largest element as the centrale and the second element as the second distal carpal. The centrale is flattened and suboval, similar to that of *Jingshanosaurus*^5^, *Lufengosaurus huenei*^2^ and *Yunnanosaurus huangi*^3^. The borders of the centrale are rugose, and both its distal and proximal surfaces are flat to slightly convex. The other carpal is angular and in contact with the proximal surface of metacarpal IV, a configuration rarely observed (Sereno^28^: Fig. 10; Goussard^29^: Fig. 12). Given its size (circa 80% of the centrale) and shape, it is most likely the second distal carpal, which articulates with metacarpal II. However, in some cases, a large carpal has been found in situ near metacarpal IV^30^. It could therefore be the fourth distal carpal, based on Läng & Goussard^27^.

The hand morphology is comparable to that of the genus *Jingshanosaurus*^5^ in terms of proportion. Based on the relative elongation of metacarpals II to V and the width over length ratio of the phalanges, the hand of *Irisosaurus* appears stouter than that of *Lufengosaurus huenei*^2^ or *Yunnanosaurus huangi*^3^. It is, however, less stout than the hand of *Yizhousaurus*^9^.

Metacarpal I is quadrangular and robust. The torsion between the proximal and distal ends is slightly marked. The distal condyles are large and dorsoventrally deep, and the distal lateral condyle projects more anteriorly than the medial condyle.

Metacarpal II is more elongated than metacarpal I. It is hourglass-shaped, with strongly concave lateral and medial margins. The distal condyles are not as prominent as on metacarpal I and are dorsoventrally lower than the proximal end of the bone.

Metacarpal III is short and appears more gracile than metacarpal II. The proximal end of metacarpal III is more extended transversely than the distal end.

Metacarpal IV is shorter than metacarpal III. Medial and lateral margins of the bone are concave, and the proximal end is markedly transversely wider than the distal end.

Metacarpal V is the shortest and the most astonishing. It is practically as wide proximally as long, curved and very stout, with concave margins. Among non-sauropodan sauropodomorphs, two shapes coexist: a subsymmetrical one like in *Yunnanosaurus huangi*^3^ and an asymmetrical one, like in *Yizhousaurus*^9^. In this specimen, the proximal half of metacarpal V is strongly asymmetrical, the proximomedial corner being much more anterior than the proximolateral corner (Fig. 10). For this reason, the proximal articular surface shows a 100° angle between the articulation with the carpus and the facet for metacarpal IV. It is the first Chinese taxon to present such a feature.

The phalangeal formula of the right manus is 2–3–(4)–?–?. Digit I is abnormally enlarged compared to the others, as in all non-sauropodan sauropodomorphs. Phalanx I.1 is robust, short, and twisted, to fit the asymmetrical shape of metacarpal I. Distally, the condyles are rather large and bear marked collateral fossae. The ginglymus is more extended ventrally than dorsally. The sharp and hooked ungual of digit I is the largest element of the manus. Non-ungual phalanges of digit II are almost as long as wide, those of digit III are longer than wide. Collateral fossae and intercondylar processes are observed on all non-ungual phalanges.

#### Pelvic girdle

##### Ischium

**Figure 11:**
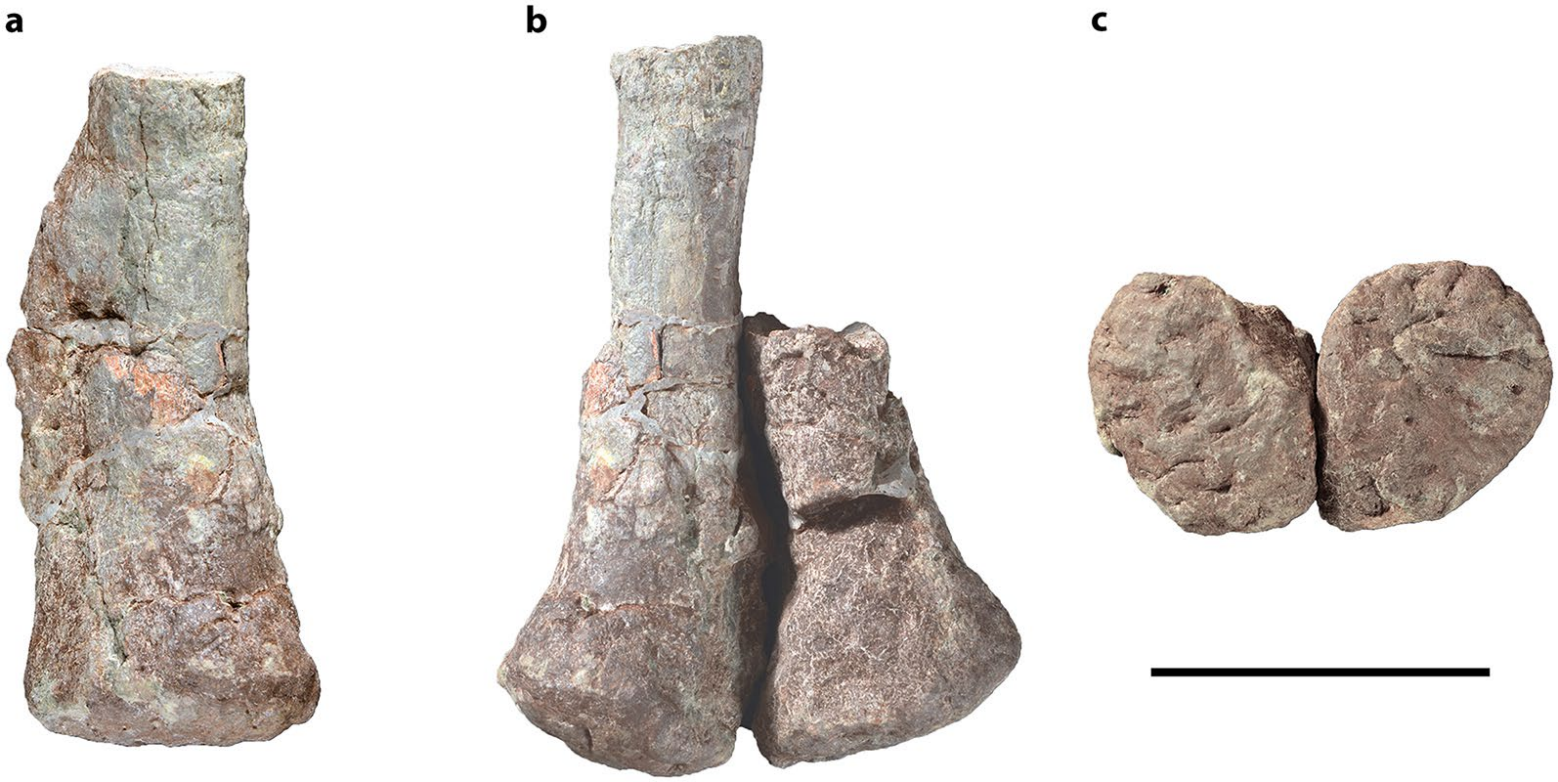
*Irisosaurus yimenensis* incomplete ischia. (**a**) Left ischium in lateral view; (**b**) Articulated ischia in dorsal view; (**c**) Articulated ischia in distal view. Scale bar = 10 cm.

The distal end of both ischia are preserved (Fig. 11; Table S5). The ischial shaft is subtriangular in cross-section, as in all non-sauropodan sauropodomorphs. The dorsal border of the shaft is thickened, whereas the ventral border is laminar. Towards the distal end of the bone, the cross-section goes from subtriangular to suboval, dorsoventrally higher than transversely wide. The dorsal border gets transversely wider distally, but the ventral border stays sharp.

The distal end of the ischium is markedly expanded dorsoventrally with respect to the shaft. In dorsal view, the lateral margin of both distal ends appears slightly concave. In lateral view, the posteroventral corner of the end is not projecting ventrally as in *Jingshanosaurus*^5^ and *Yunnanosaurus youngi*^25^. In distal view, ischial ends have a subcircular outline. This is also observed in in *Xixiposaurus*^7^, whereas other Chinese genera, such as *Lufengosaurus*^2^ or *Yunnanosaurus*^3,25^ have a more suboval outline.

#### Hindlimb

##### Pes

Only one partial left ungual phalanx is preserved. It is curved and transversely compressed, with the nutrient groove visible on the lateral surface, but not on the medial surface. The dorsal process and flexor tubercle are prominent. The proximal articular surface is strongly concave dorsoventrally, suboval in outline, and is subdivided into two depressions by a median crest.

## Discussion

Despite the incompleteness of the material, *Irisosaurus* is clearly a non-sauropodan sauropodomorph dinosaur as indicated by numerous anatomical characteristics: folidont teeth without occlusion surface; vertebrae with poorly developed laminae; phalanx I on manual digit I bearing a proximal “heel” and phalanx I.1 having a twisted long axis^1^. These features are present in most known non-sauropodan sauropodomorphs, although earliest-diverging or relatively-late-diverging forms show some variation. Based on the preserved material, *Irisosaurus* has a body plan close to that of the so-called “core prosauropods”^28^ in having, for instance, cervical vertebrae longer than most dorsal vertebrae, gracile forelimbs, a deltopectoral crest extending half of the total length of the humerus and a unique carpal-metacarpal complex.

**Figure 12:**
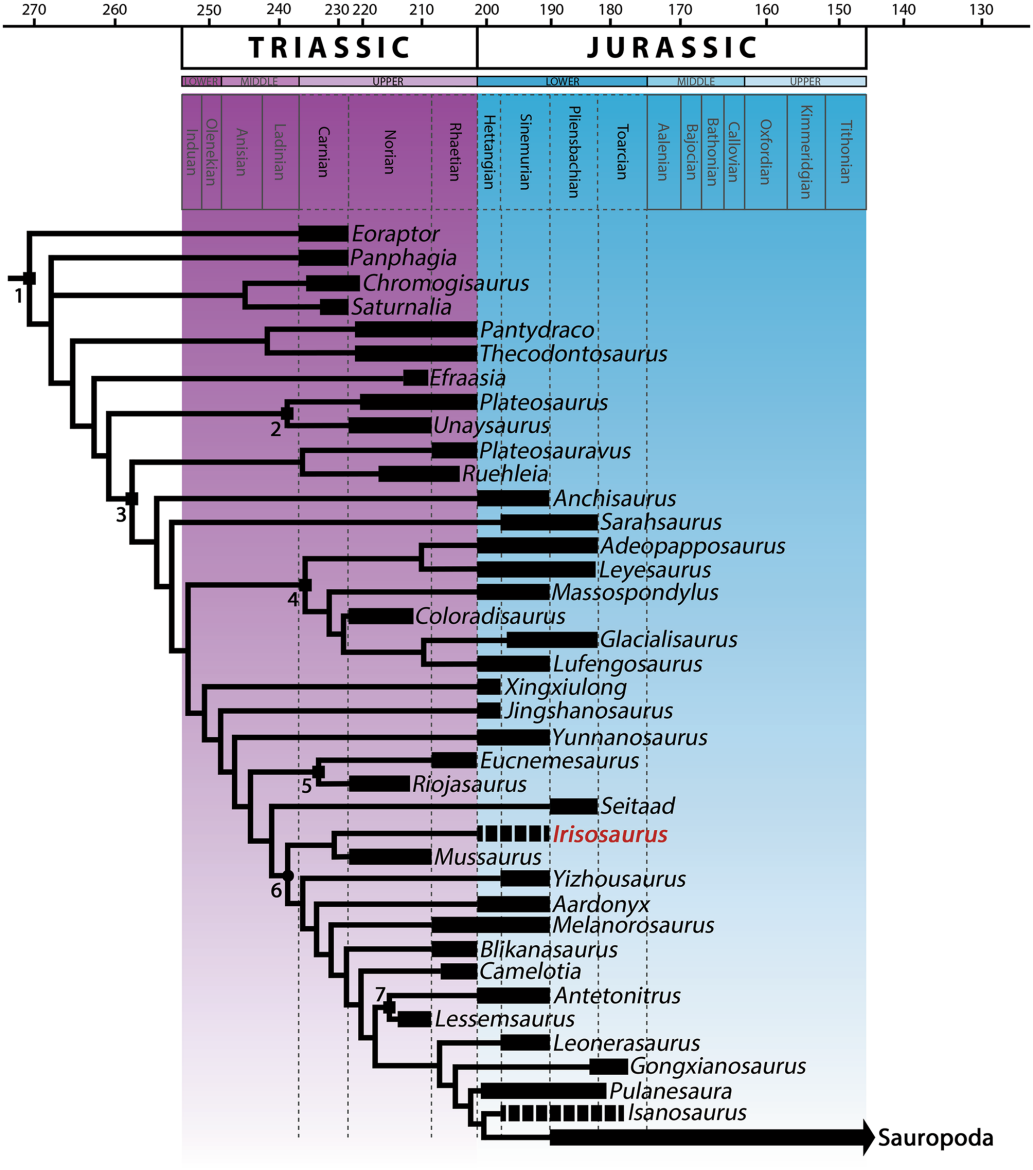
Strict reduced consensus at the genus level of the most parsimonious trees recovered following the phylogenetic analysis. The original dataset is based on the matrix and scorings of Zhang et al. 2019 (except for *Irisosaurus*) and includes 62 taxa for 364 characters. This reduced consensus displays 39 taxa. It is based on 2 MPTs of 1,300 steps each (CI = 0.33, RI = 0.69). Dashed lines mean that the exact age of the genus is uncertain. Clades: 1, Sauropodomorpha; 2, Plateosauridae; 3, Massopoda; 4, Massospondylidae; 5, Riojasauridae; 6, Sauropodiformes; 7, Lessemsauridae. Squares represent stem-based definitions, ellipses represent node-based definitions.

We carried out a comprehensive cladistic analysis based on a matrix consisting of 364 characters and 62 terminal taxa. Following this analysis, we produced a strict consensus tree (Fig. 12), where *Irisosaurus* is recovered as an early-diverging sauropodiforme, sister-taxon of the Triassic *Mussaurus*. The basalmost part of the phylogenetic tree displays two clades including *Chromogisaurus* and *Saturnalia* on one side and the juvenile *Pantydraco* and *Thecodontosaurus*, on the other side. The clade Plateosauridae follows, before the first dichotomy in Massopoda, where a clade regrouping *Ruehleia* and *Plateosauravus* is recovered. The family Massospondylidae is positioned in a more “apical” (i.e., close to Sauropoda) position and the branches following Massospondylidae bear consecutively three genera from the Lufeng Formation of China: *Xingxiulong*, *Jingshanosaurus* and *Yunnanosaurus*. Riojasauridae are recovered more apically than the previous genera, but more basally than the clade comprising *Irisosaurus* together with *Mussaurus*. Both clades are separated by one taxon: the north American *Seitaad*. Apically to early-diverging Sauropodiformes, the Chinese genus *Yizhousaurus* is recovered, followed by several massive ‘sauropod-like’ forms, such as Lessemsauridae, *Gongxianosaurus* or *Pulanesaura*. *Isanosaurus* is recovered as the sister-taxon of Sauropoda.

The phylogenetic analysis confirmed that *Irisosaurus* belongs to non-sauropodan sauropodomorphs. A combination of plesiomorphic and apomorphic features places this new genus among Sauropodiformes, between early-branching (“core prosauropods”) and late-branching (“sauropod-like”) members of non-sauropodan sauropodomorphs. To investigate this result, we considered unambiguous changes along branches. Sauropodiformes are diagnosed by two unambiguous synapomorphies: minimum transverse shaft width of metacarpal I less than twice the minimum transverse shaft width of metacarpal II (character 229, state 0) and position of the lateral margin of the descending posteroventral process of the distal end of the tibia set well back from the anterolateral corner of the distal tibia (character 315, state 1). The clade including *Mussaurus* and *Irisosaurus* is diagnosed by only one unambiguous synapomorphy: humeral head slightly-developed and rounded in anteroposterior view (character 209, state 0).

**Figure 13:**
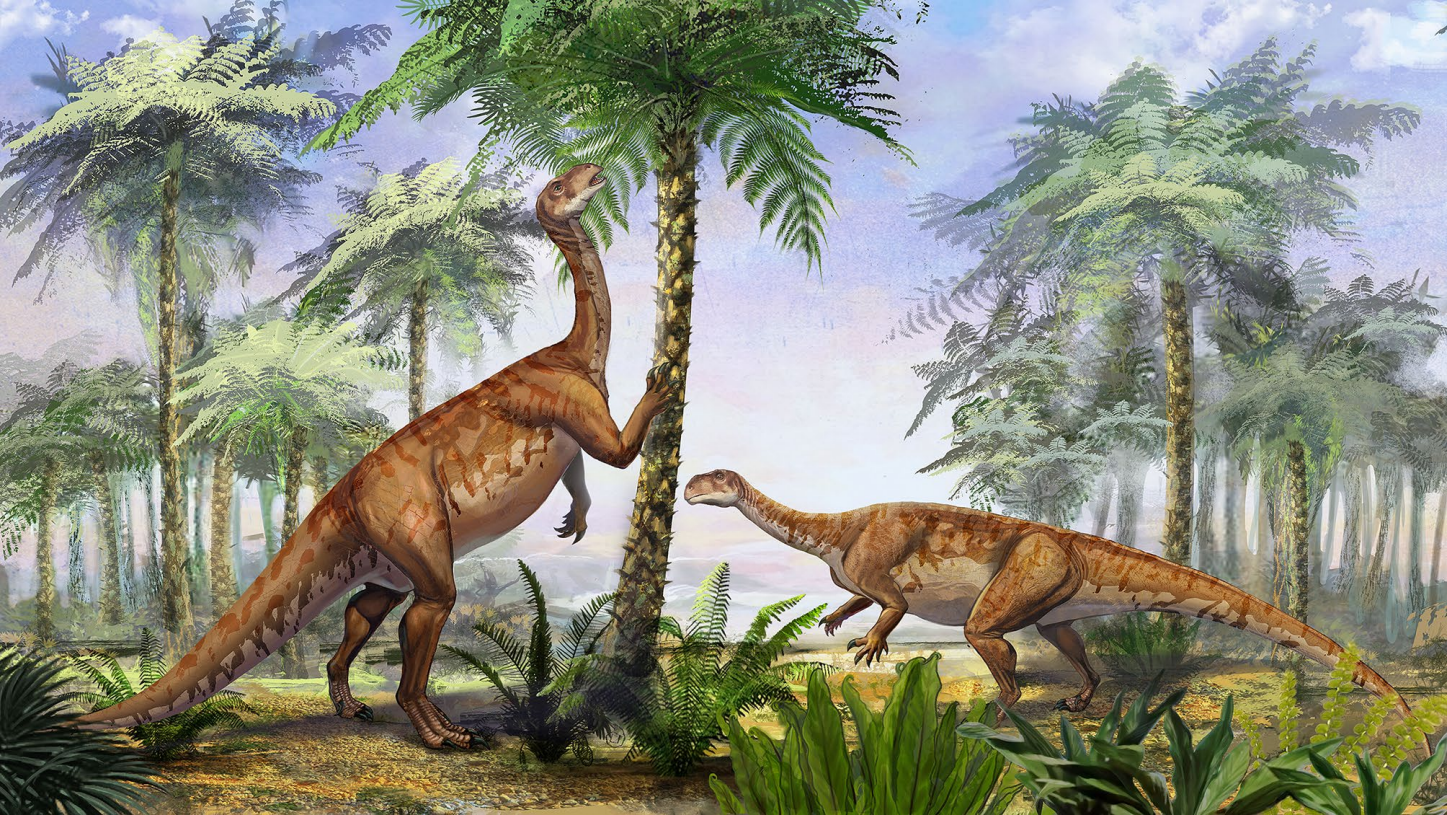
*Irisosaurus yimenensis* life reconstruction, by Ang Li.

The recovery of *Irisosaurus* among Sauropodiformes is a slightly surprising result. Based on the postcranial anatomy of *Irisosaurus*, particularly its forelimb which is not adapted for locomotion, we consider it biped (Fig. 13). This is consistent with the fact that most non-sauropod sauropodomorphs are believed to be either facultative quadrupeds or bipeds^31^. Still, several genera recovered near *Irisosaurus* in the phylogenetic tree commonly present robust femora, such as *Riojasaurus* and *Yizhousaurus*, or were regarded as the oldest quadruped forms before Sauropoda, as was the case for *Aardonyx*^32^. However, the once “noticeable craniolateral process” of the ulna in the latter genus is observed in a number of specimens, including *Irisosaurus*, such as *Mussaurus*, *Yizhousaurus* or even *Yunnanosaurus*, that do not display further adaptations to quadrupedalism. *Irisosaurus* forelimb is comparable to that of its sister-taxon *Mussaurus*, although bulkier. Following Otero et al.^31^, it is then most likely that the mobility of *Irisosaurus* manual digit I was important, including for non-locomotor behaviors such as grasping. Additionally, passive and full manus pronation was presumably not present at the base of Sauropodiformes, although it was hypothesized that some small amount of active pronation of the manus was possible in *Mussaurus* and, therefore, in close relatives. This pronation was seemingly made possible via long-axis rotation at the elbow to achieve semi-pronation of the whole antebrachium (not rotation of the radius around the ulna, as previously thought)^31^.

Despite being recovered in the same clade, the laurasian *Irisosaurus* and the Gondwanian *Mussaurus* show numerous anatomical discrepancies: *Mussaurus* has a maxilla with a row of six neurovascular foramina on its lateral surface, an anteroposteriorly elongate premaxillary ramus, and a reduced perinarial fossa with no deep neurovascular foramen within^33^. *Mussaurus* also has a scapula with the proximal end more expanded than the distal end, a ventromedial robust ridge reaching the distal third of the scapular blade, as well as a gracile forelimb with elongated metacarpals^34^.

*Irisosaurus* is the second occurrence, after *Yunnanosaurus huangi,* of a species with straight and mesiodistally compressed tooth crowns lacking denticles. It has been reported that some of the *Y. huangi* material exhibited apical denticles^14^, but this feature is not observed on the few well-preserved teeth of *Irisosaurus*. This absence of denticles, which was exclusive to *Y. huangi* for a long time, was interpreted as the result of a dietary specialization^14^. We know now that this specialized herbivory was at least shared with *Irisosaurus*. These two genera followed a similar pattern in terms of teeth evolution, maybe as a result of environmental stress induced by the myriad of non-sauropodan sauropodomorph species occupying southern Asian ecosystems during Early Jurassic. The general pattern in non-sauropodan sauropodomorphs from the Lufeng fauna is coarsely serrated teeth, which are associated with herbivory to opportunistic omnivory^1^. The denticle loss in *Yunnanosaurus* and *Irisosaurus* can be interpreted as an evolutionary trend to adapt to a specific kind of vegetation and therefore occupy a different ecological niche than the bulk of sauropodomorph populations.

The Fengjiahe Formation, deposited between the Upper Triassic Shezi Formation below and the Middle Jurassic Zhanghe Formation above^13^, is rich in vertebrate fossils. It yielded non-sauropodan sauropodomorphs originally reported from the Lufeng Formation, such as *Lufengosaurus* and *Yunnanosaurus*^12^, although it is stratigraphically slightly older than the abovementioned Formation^13^. Yet, most probably because of a sampling bias, only two sauropodomorph genera were exclusively recovered in the Fengjiahe Formation in the past. *Chinshakiangosaurus* was collected in the 1970s and identified as a primitive sauropod based on a lower jaw and postcranial material^11^. It was never properly published and is therefore a nomen nudum. *Yimenosaurus* was found in 1987 and erected in 1990 based on several skeletons discovered near the type locality of *Irisosaurus*^4^. Although *Yimenosaurus* has never been included in recent cladistic analyses^6–9^, it was originally described as a Plateosauridae, and thus is relatively distantly related to *Irisosaurus*. However, given that these two taxa are both from the Fengjiahe Formation of the same geographical area, we present below detailed comparisons. Because we were not granted permission to examine the *Yimenosaurus* specimens to collect first-hand data, we had to use the information from the original publication for comparisons. Neverthless, our comparisons demonstrate that the two taxa are clearly different from each other, probably representing two relatively distantly placed lineages on sauropodomorphan trees. The most obvious difference between *Irisosaurus* and *Yimenosaurus* is the larger body size of *Yimenosaurus* (length is estimated around 9 m, versus 5 m for *Irisosaurus*). The maxilla of *Yimenosaurus* has a relatively long premaxillary ramus, while it is relatively short in *Irisosaurus*. The premaxillary ramus does not bear any structure in *Yimenosaurus*, while it bears a ridge associated to a deep perinarial fossa including a large foramen in *Irisosaurus*. The premaxillary articulation surface is dorsally developed and square-shaped in *Yimenosaurus*, while its preserved part in *Irisosaurus* is less developed dorsally (making it unlikely to have a square process). The maxilla nasal process is particularly gracile with a thin base in *Yimenosaurus*, while the base is large in *Irisosaurus*. Near the dental margin, the maxilla lateral surface of *Yimenosaurus* shows a series of irregularly sized nutrient foramina, unlike *Irisosaurus*. The maxillary dentition has denticles in *Yimenosaurus*, while they are absent in *Irisosaurus*. *Yimenosaurus* axis has an important length over height ratio, and proportions of the axis centrum are always retained along most of the cervical series. Thereby, anterior to middle cervical vertebrae of *Yimenosaurus* share a high length over height ratio, while corresponding vertebrae in *Irisosaurus* have a much lower ratio, due to the short and tall morphology of centra.

Despite an already high diversity of early-branching sauropodomorphs in the Early Jurassic of China, this study reveals yet another new species, adding to the known dinosaurian diversity and highlighting an unprecedented evolutionary mechanism that would have led some Chinese forms to a specialized herbivorous diet.

## Methods

### Terminology

Traditional anatomical and directional terms are employed in this manuscript, rather than their veterinarian alternatives^35^. For instance, “anterior” and “posterior” are used as directional terms instead of the veterinarian “cranial” or “rostral” and “caudal”. We follow Wilson^36^ regarding the identification of vertebral laminae.

For Basal Sauropodomorpha, the phylogenetic definitions taken into consideration are those proposed by Sereno^28^. However, the taxon Massopoda having been erected simultaneously to the publication of Sereno’s review^28^, we follow here the original definition given by Yates^37^. Sauropoda is defined following the node-based definition of Salgado et al.^38^.

### Phylogenetic analysis

To investigate the systematic position of *Irisosaurus*, we conducted a phylogenetic analysis based on a modified version of the dataset of Zhang et al.^17^. The original dataset has 61 taxa and 364 characters. Following Zhang et al.^17^, 43 multistate characters (8, 13, 19, 23, 40, 57, 69, 92, 102, 117, 121, 131, 134, 145, 148, 150, 151, 158, 163, 168, 171, 178, 185, 208, 211, 218, 226, 231, 238, 246, 254, 257, 270, 282, 303, 309, 317, 337, 350, 353, 355, 360, 364) were considered additive. We added *Irisosaurus* to operational taxonomic units and did not change the number of characters or their scorings. The current matrix has 62 taxa and 364 characters. 80 characters were scored for *Irisosaurus*, that is 22% of the total number of characters.

The phylogenetic analysis was runned using TNT version 1.5^39^ (https://www.lillo.org.ar/phylogeny/tnt/). The traditional search method was employed, with 1,000 Wagner trees replicates of additional sequences and a tree bisection reconnection (TBR) swapping algorithm with 10 trees per replication. The analysis resulted in 2 most parsimonious trees, each with a length of 1,300 steps, a consistency index of 0.33 and a retention index of 0.69.

## Supplementary information

Supplementary file1
