# Author Correction: A new species of early-diverging Sauropodiformes from the Lower Jurassic Fengjiahe Formation of Yunnan Province, China

**DOI:** 10.1038/s41598-020-74208-4

**Published:** 2020-10-06

**Authors:** Claire Peyre de Fabrègues, Shundong Bi, Hongqing Li, Gang Li, Lei Yang, Xing Xu

**Affiliations:** 1 Centre for Vertebrate Evolutionary Biology, Institute of Palaeontology, Yunnan University, Kunming, 650091 China; 2 Department of Biology, Indiana University of Pennsylvania, Indiana, PA 15705 USA; 3 Yimen Administration of Cultural Heritage, Yimen, 651100 China; 4 Key Laboratory of Evolutionary Systematics of Vertebrates, Institute of Vertebrate Paleontology and Paleoanthropology, Chinese Academy of Sciences, Beijing, 100044 China; 5 Center for Excellence in Life and Paleoenvironment, Beijing, 100044 China

Correction to: *Scientific Reports* 10.1038/s41598-020-67754-4, published online 03 July 2020

Peyre de Fabrègues* et al*. (2020), which was published electronically, does not include evidence of registration in ZooBank within the work itself, which is a requirement by Article 8.5.3 of the International Code of Zoological Nomenclature^1^. Therefore, the newly proposed species-group name *Irisosaurus yimenensis* is not available.

This publication has been registered in ZooBank with the LSID: urn:lsid:zoobank.org:act:462C8657-095A-41F2-AF81-E0F28185ABAC. The following ‘Zoobank’ and ‘Nomenclatural Acts’ subsections appear below, along with the ‘Systematic Paleontology’ section of the original Article^2^. In addition, the ‘Etymology’ subsection has been corrected below to reflect the origin of the species-group name.

**Systematic Paleontology.**

- Dinosauria Owen, 1842
- Saurischia Seeley, 1887
- Sauropodomorpha von Huene, 1932
- Massopoda Yates, 2007
- Sauropodiformes Sereno, 2007
- *Irisosaurus yimenensis* gen. et sp. nov.

**Zoobank.**

- urn:lsid:zoobank.org:act:462C8657-095A-41F2-AF81-E0F28185ABAC.

**Etymology.**

- The generic nomen, from the latin iris (rainbow, reflections and by extension, iridescent), refers to the famous iridescent clouds of Yunnan Province (彩云之南). The specific epithet refers to Yimen County, where the type locality is located.

**Nomenclatural Acts.**

- The electronic version of this article in Portable Document Format (PDF) will represent a published work according to the International Commission on Zoological Nomenclature (ICZN), and hence the new names contained in the electronic version are effectively published under that Code from the electronic edition alone. This published work and the nomenclatural acts it contains have been registered in ZooBank, the online registration system for the ICZN. The ZooBank LSIDs (Life Science Identifiers) can be resolved and the associated information viewed through any standard web browser by appending the LSID to the prefix https://zoobank.org/. The LSID for this publication is: [urn:lsid:zoobank.org:pub:5E157E76-19E1-48D9-AD7A-A1302CF81D96]. The online version of this work is archived and available from the following digital repositories: PubMed Central and CLOCKSS.
