# Supplementary material for: A new species of early-diverging Sauropodiformes from the Lower Jurassic Fengjiahe Formation of Yunnan Province, China
Source: Sci Rep. 2020 Jul 3;10:10961. doi: 10.1038/s41598-020-67754-4 (PMC7335049; doi:10.1038/s41598-020-67754-4)
Supplement: Supplementary file 1 — Supplementary file1 [file 41598_2020_67754_MOESM1_ESM.docx]

**SUPPLEMENTARY INFORMATION**

**A new species of early-diverging Sauropodiformes from the Lower Jurassic Fengjiahe Formation of Yunnan Province, China**

**Claire Peyre de Fabrègues**^1, *^**, Shundong Bi^2,^**^1^**, Hongqing Li**^1^**, Gang Li**^1^**, Lei Yang^3^, Xing Xu**^4,5, *^

^1^ Centre for Vertebrate Evolutionary Biology, Yunnan University, Kunming, 650091, China

^2^ Department of Biology, Indiana University of Pennsylvania, Indiana, PA, 15705, USA

^3^ Yimen Administration of Cultural Heritage, Yimen, 651100, China

^4^ Key Laboratory of Evolutionary Systematics of Vertebrates, Institute of Vertebrate Paleontology & Paleoanthropology, Chinese Academy of Sciences, Beijing, 100044, China

^5^ Center for Excellence in Life and Paleoenvironment, Beijing, 100044, China

^*^ Correspondence and requests for materials should be addressed to [claire.pdf@gmail.com](mailto:claire.pdf@gmail.com) or [xu.xing@ivpp.ac.cn](mailto:xu.xing@ivpp.ac.cn)

Data S1. Anatomical description & Comparisons

Figure S1. Photographs of left scapular girdle and forelimb

Table S1. Measurements of vertebrae

Table S2. Measurements of scapulae

Table S3. Measurements of coracoids

Table S4. Measurements of forelimbs

Table S5. Measurements of ischia

Figure S2. Strict consensus tree

Data S2. Phylogenetic matrix

Data S3. 3D model of the maxilla

**Supplementary Data S1**

Detailed anatomical description

#### **Skull.**

##### ***Maxilla***

When complete, the maxilla is a triradiate bone which forms the posterior and ventral margins of the naris and the ventral and anterior margins of the antorbital fenestra. Here, the left maxilla consists of the anterior part of the main tooth-bearing body overhung by the broken base of the nasal process and of a fragment of the posterior ramus (Figs. 4 & 5).

The lateral surface of the maxilla is smoothly convex dorsoventrally and does not bear a lateral maxillary ridge, like in *Lufengosaurus*^1^. Only two very small neurovascular foramina are visible on the lateral surface of the premaxillary ramus, and none is observed on the fragment of the posterior ramus. This is a very surprising feature, given that most Jurassic non-sauropodan sauropodomorphs in which the maxilla is known have numerous (4 to 8) large neurovascular foramina, most of which are located on the anterior portion of the maxilla (Fig. 4). The closest condition to that of *Irisosaurus* has been described in *Yunnanosaurus huangi*^2^, which complete absence of foramina is considered an autopomorphy.

The portion of the premaxillary ramus prior to the nasal process is short, as in *Lufengosaurus*^1^ and *Pradhania*^3^, while it is proportionally shorter in *Yunnanosaurus*^2,4^. It is also markedly expanded dorsoventrally relative to its anteroposterior development. In most other genera, such as *Jingshanosaurus*^5,6^, *Lufengosaurus*^1^, *Xixiposaurus*^7^, *Yimenosaurus*^8^ and *Yizhousaurus*^9^, the premaxillary ramus is longer than high. The anterior articulation surface of the ramus is complete. On its anterodorsal portion, the base of the premaxillary articular surface is visible. It is strongly deflected medially.

The premaxillary ramus of the maxilla bears an elongate curved ridge delimiting the posteroventral corner of the perinarial fossa. Only two other Asian taxa (*Jingshanosaurus*^5,6^ and *Xixiposaurus*^7^) display a quite elongate ridge. The extension of the perinarial fossa on the maxilla is present in most non-sauropodan sauropodomorphs. It is observed in several Chinese forms in which the skull is preserved: *Jingshanosaurus*^5,6^, *Lufengosaurus*^1^, *Xixiposaurus*^7^, *Yizhousaurus*^9^ and *Yunnanosaurus* (pers. obs., ZLJ0110). Along the ridge, the perinarial fossa incorporates a large and deep neurovascular foramen opening anteriorly. This feature is not present in any other early-diverging sauropodomorph for which appropriate material is known. We therefore consider it an autapomorphy of *Irisosaurus*.

Dorsally, the broken base of the nasal process is directed posterodorsally. Posterior to this base, the maxilla bears a marked concavity corresponding to the anteroventral corner of the antorbital fenestra. Surprisingly, and conversely to what is observed in most non-sauropodan sauropodomorphs, no antorbital fossa (associated with the presence of a medial lamina) is visible. To our knowledge, this feature is not observed in any other genus, except maybe *Pradhania*, for which it is not specified in the description^3^. On the jugal ramus of the maxilla, a deep dorsally opening groove separates the lateral surface of the bone from the medial tooth-bearing part. It most likely helped to accommodate the lachrymal.

The medial surface of the partial maxilla appears flat, except for the anterodorsal inflexion corresponding to the premaxillary articular surface. Along the alveolar margin, eight alveoli are preserved on the anterior part of the maxilla, and four on the posterior ramus fragment. Five alveoli show replacement teeth preserved *in situ*. The teeth lie higher than the alveolar margin, therefore the toothrow is exclusively visible in medial view.

##### ***Dentary***

The dentary is a thick and elongate element of the mandible, here only represented by a small fragment from the right side. The dorsal margin of the preserved part diverges from the ventral one, so that the posterior dorsoventral depth of the fragment is approximately twice its anterior dorsoventral depth. The medial surface of the dentary fragment bears the last tooth alveoli. Their presence, coupled to the aforementioned proportions, allow to identify the fragment as the posteriormost part of the dentary, close to the articulation with the surangular and angular. The lateral surface of the dentary is flat, while the ventral surface shows what seems to be the posterior end of the Meckelian groove.

##### ***Dentition***

Maxillary teeth appear quite robust, but all of them are damaged and not completely erupted. Besides these, three isolated teeth were recovered. All of them are subcomplete, but damaged at varying degrees. The best-preserved one has a complete crown and base of the root (Fig. 5).

The teeth are straight folidont, labiolingually compressed and with a reniform cross-section^10^. The crown is spatulate, with a strongly convex labial surface and a smoothly concave lingual surface, closer to what is observed in *Lamplughsaura*^3^, *Yunnanosaurus*^2,4^, *Leonerasaurus*^11^, and *Pulanesaura*^12^, than in other non-sauropodan sauropodomorphs. The mesiodistally widest point of the crown is not located at the base of the crown, like in most non-sauropodan sauropodomorph genera, but at mid-height. This condition is also observed in *Jingshanosaurus*^5,6^, *Lamplughsaura*^3^, *Leonerasaurus*^11^ and *Yunnanosaurus*^2,4^. However, compared to the root, the mesiodistal expansion is not much pronounced. The Slenderness Index (‘SI’: length of the tooth crown divided by its maximum mesiodistal width^13^) of *Irisosaurus* is 2.36, that is higher than *Lufengosaurus* (1.70) and *Yunnanosaurus* (1.95), but lower than *Yimenosaurus* (2.43)^14^.

The carinae do not bear denticles, conversely to the condition of most non-sauropodan sauropodomorphs except *Yunnanosaurus huangi*^2^. At the apex of the crown, no wear facets are visible.

#### **Axial skeleton.**

##### ***Cervical Vertebrae***

Five vertebrae, more or less complete, were identified as cervical vertebrae (Fig. 6; Table S1). They are elongated, with no marked pneumatization visible on the centra and neural arches. The cervical centra are all amphicoelous with relatively flat lateral surfaces. They are subequal in length and the small variations in size follow the pattern observed in most early-diverging sauropodomorphs, where the longest cervical vertebrae are usually in the middle of the series (C5 or C6) and the shortest (without considering atlas and axis) are the last ones (C9 and C10). The longest middle cervical centrum is 1.9 times longer than high on its anterior surface. This ratio is among the lowest obtained for a non-sauropodan sauropodomorph, meaning either that *Irisosaurus* has relatively low cervical vertebrae or that the preserved middle centrum is not the longest of the series. These proportions are similar to those of the longest cervical vertebra of *Lamplughsaura*^3^ and *Yizhousaurus*^9^ (ratios of 2.0), and are close to those calculated for *Lufengosaurus*^15,16^, *Xingxiulong*^17^, *Yunnanosaurus huangi*^4^ and *Yunnanosaurus youngi*^18^ (ratios of 2.4). Otherwise, most non-sauropodan sauropodomorphs have a length over height ratio between 3 and 4.5, that is proportionally longer cervical centra. Cervical centra have subcircular articular surfaces and, on their ventral aspect, they all present a median constriction. The most anterior middle cervical seems to bear an incipient ventral ridge throughout its length, like in *Lufengosaurus huenei*^15^, *Xingxiulong*^17^ or *Yizhousaurus*^9^, while the more posterior middle cervical centrum has a clear ventral keel on its anterior half. Both posterior cervical vertebrae exhibit a small hypapophysis merged with a short anterior ventral keel.

Parapophyses are hardly visible on the anterior cervical vertebra. On middle cervical vertebrae they are low, reduced in size and located in the anteroventral corner of the centrum. On the first one, parapophyses are not well defined whereas, on the second one, they are better defined, rounded and tubercle-like. On posterior cervical vertebrae, parapophyses are large, rounded tubercles overlapping the anterior margin of the centrum.

Diapophyses are incipient, non-projecting, subtriangular processes on the anterior cervical vertebra, like in most non-sauropodan sauropodomorphs. On the middle cervical vertebra they are more visible, and rise at the level of the neurocentral suture, in the anterior half of the vertebra. On the posterior cervical vertebra, the diapophysis is distinctly more developed and located more posterodorsally on the vertebra. It is directed lateroventrally, with a strong ventral component. The diapophysis is subtriangular in shape, and appears quite similar to what is observed on the 8^th^ cervical of *Yunnanosaurus youngi*^18^. On either side of it, two blunt laminae can be identified: the anterior centrodiapophyseal lamina (acdl) and the posterior centrodiapophyseal lamina (pcdl).

Most of the cervical neural arches are not preserved. On one posterior cervical vertebra, a prezygodiapophyseal lamina (prdl) is visible although prezygapophyses were not preserved. Posteriorly, the preserved left postzygapophysis is projecting laterodorsally and has a flat articular surface. This laterodorsal projection is generally occuring at the end of the cervical series, although it is observed in the middle cervical vertebrae of *Lamplughsaura*^3^. On the lateral surface of the neural arch, a marked postzygodiapophyseal lamina (podl) is visible. In posterior aspect, an intrapostzygapophyseal lamina (tpol) and a spinopostzygapophyseal lamina (spol) are observed. The postzygapophysis does not bear any epipophysis on its dorsal surface, conversely to postzygapophyses of *Yizhousaurus*^9^ and *Yunnanosaurus youngi*^18^.

The neural arch below the neural spine is low on anterior and middle cervical vertebrae. The neural spine base is approximately 50 mm long, and appears transversely compressed on the middle cervical vertebra. On the posterior cervical vertebra, the neural arch below the neural spine is high. The base of the neural spine is shorter, approximately 40 mm long, and transversely thick. The bulging neural spine is drop-shaped in dorsal aspect. In lateral view, it is quadrangular as in *Xingxiulong*^17^ C9 and *Yizhousaurus*^9^ C10.

##### ***Dorsal Vertebrae***

One incomplete vertebra, one isolated neural spine and one centrum were identified as dorsal vertebrae (Fig. 7; Table S1). Centra are higher and shorter than in cervical vertebrae. They are amphicoelous, robust and subequal in length. Shallow lateral depressions are observed on centra, but no marked pneumatization. Broadly speaking, in early-diverging sauropodomorphs the first dorsal centrum has a length/height ratio circa 1.3 (mean based on 10 different specimens), ratio found as well in *Irisosaurus* anterior dorsal vertebra and in *Yizhousaurus*^9^ D1. It is close to that of *Xingxiulong*^17^ D1 (1.2), superior to that of *Lufengosaurus magnus*^16^ D1 and *Yunnanosaurus youngi*^18^ D1 (1), and inferior to that of *Yunnanosaurus robustus*^19^ D2 (1.6). Both dorsal centra have subcircular articular surfaces and, in ventral aspect, a marked median constriction. The anterior centrum ventral surface exhibits a hypapophysis and a marked ventral keel on its entire length. Ventral keels have also been described on the anterior dorsal vertebrae of *Lufengosaurus huenei*^15^, *Xingxiulong*^17^, *Yizhousaurus*^9^ and *Yunnanosaurus*^4,18^, among others.

Parapophyses of the anterior dorsal vertebra are oval-shaped and low. They are located in the dorsomedian area of the centrum, where they overlap the neurocentral suture, as in most non-sauropodan sauropodomorph taxa. On the middle dorsal centrum, parapophyses are not visible and have therefore completely shifted to the neural arch.

Diapophyses from the dorsal series are only preserved among isolated vertebra fragments. They are more developed than those of the posterior cervical vertebra and project laterally. They are quadrangular in dorsal view, while several unidentified laminae and associated fossae are visible on the ventral aspect. On the anterior dorsal vertebra, the diapophyses base is bordered posteroventrally by a posterior centrodiapophyseal lamina (pcdl), and by an anterior centrodiapophyseal lamina (acdl) anteroventrally.

The isolated dorsal neural spine preserves both postzygapophyses, one being still articulated with the corresponding prezygapophysis. Articular surfaces are flat, circular and oriented strictly dorsally and ventrally for the prezygapophysis and postzygapophyses, respectively. In posterior view, the postzygapophyses orientation has a small lateral component. Above postzygapophyses, small spinopostzygapophyseal laminae (spol) are visible on the posterior aspect. The dorsal surface of postzygapophyses does not bear any epipophysis.

The neural arch to vertebra height ratio of the anterior dorsal vertebra is low in comparison with the posterior cervical vertebra. An anterior dorsal isolated neural spine has a base as long as in the posterior cervical vertebra, but a high and subrectangular shape in lateral view. Few Chinese early-diverging sauropodomorphs preserve neural spines around the cervicodorsal transition, but of these, *Xingxiulong*^17^ is the one with the closest anatomy. In dorsal view, the isolated neural spine is transversely wider than anteroposteriorly long, a condition commonly observed in the first two dorsal vertebrae of non-sauropodan sauropodomorphs. It is bulging and drop-shaped, like on the posterior cervical vertebra, but with a distal end more expanded in lateral view relatively to the base of the neural spine. On the anterior dorsal vertebra, the preserved part of the neural spine is much longer than wide and transversely compressed. The middle isolated neural spine is also elongated and compressed. This is typically observed between D5 and D10, the neural spines usually becoming shorter and higher after D10, towards the sacrum.

#### **Pectoral Girdle.**

Both scapulae and coracoids were preserved and fused together. The right scapula and coracoid were separated during the preparation of the specimen (Fig. 8; Tables S2 & S3). The left scapulocoracoid has several damaged or missing parts, which have been covered by plaster (Fig. S1; Tables S2 & S3).

For description and measurements, scapulocoracoids are considered with the long axis positioned vertically. Following this orientation, the coracoid is ventral, the acromion is anterior and the glenoid is posterior. We assume that, in vivo, the scapulocoracoid was oriented obliquely with respect to the longitudinal axis of the animal, with the coracoid located anteroventrally.

The scapula is elongate and slender, like in *Lufengosaurus*^15^, *Xixiposaurus*^7^ and *Yunnanosaurus huangi*^4^. As in all non-sauropodan sauropodomorphs, the proximal and distal ends of the scapula are anteroposteriorly extended with respect to the blade. The distal end is fan-shaped, and the damaged posterodorsal corner of the scapula looks like it was projecting further than the posteroventral corner, like in *Jingshanosaurus*^5^, *Yizhousaurus*^9^ or *Yunnanosaurus huangi*^4^.

The scapular blade (above the glenoid, without the distal end) represents circa 50% of the total length of the bone. The lateral margin of the blade is dorsoventrally convex and its medial margin is dorsoventrally concave, like in most non-sauropodan sauropodomorphs. In lateral view, the anterior and posterior margins of the scapular blade are subparallel on most of their length, but concave when considering their proximal and distal ends. This condition is also observed in several Chinese taxa, such as *Jingshanosaurus*^5^, *Lufengosaurus*^15,16^, *Yizhousaurus*^9^ or *Yunnanosaurus huangi*^4^. Anteroposteriorly, the minimal width of the blade equals 40% of the maximal anteroposterior extension of the proximal end of the scapula. Transversely, at midlength, the width of the blade represents 39% of the transverse width of the scapula at the level of the glenoid. In transverse section, the blade has an oval shape. The lateral and medial surfaces of the blade are smooth, but the medial one bears a blunt posteroventral ridge following the main axis of the bone that is most probably surrounding a muscle attachment area.

The proximal end of the scapula shows almost the same anteroposterior extension than its distal end. In some taxa, like *Lufengosaurus huenei*^15^, the proximal end is clearly more extended than the distal end. The proximal end is subrectangular, with an anteroposterior length clearly superior to the dorsoventral height. The shallow acromion fossa extends on most of it. The acromion is not projecting much anteriorly and its minimal height, measured on the anterior margin, represents 18% of the complete scapula. The dorsal margin of the acromion is oblique, slightly concave and without visible angle, as in *Jingshanosaurus*^5^, *Yizhousaurus*^9^ or *Yunnanosaurus* *huangi*^4^. Conversely, in *Lufengosaurus*^15,16^, a distinct angle is visible between the scapular blade and the acromion. The dorsal margin of the acromion is positioned at an angle of 120° to the main (dorsoventral) axis of the scapula. As often in non-sauropodan sauropodomorphs, the ventral part of the acromion is the transversely thinnest part of the scapula. Opposite to the acromion, the glenoid occupies the posterior margin of the scapula proximal end and represents the transversely thickest part of the bone. The glenoid facet is subcircular, and the sharp posterodorsal corner of the glenoid projects posteriorly. A similar degree of projection is observed in *Lufengosaurus huenei*^15^, *Yizhousaurus*^9^ and *Yunnanosaurus* *huangi*^4^. In contrast, the projection is not as important in *Jingshanosaurus*^5^ and *Lufengosaurus magnus*^16^. The ventral margin of the scapula is sigmoid.

The coracoid is a robust, oval-shaped bone, showing little morphological variation within non-sauropodan sauropodomorphs. The coracoid height over length ratio equals 73%, like in *Yizhousaurus*^9^. This value is one of the highest among non-sauropodan sauropodomorphs, meaning that the coracoid is particularly developed dorsoventrally. In comparison, the ratio in *Jingshanosaurus*^5^ and *Lufengosaurus*^15,16^ is approximately 60%. The transversely thinnest part of the coracoid is located on its anterior part whereas, like on the scapula, the thickest part is the glenoid cavity. The medial surface of the coracoid is concave, apart from a dorsal protrusion, while the lateral surface is slightly convex. It bears a laterally projecting coracoid tubercle, feature observed in all non-sauropodan sauropodomorphs with varying degrees of projection. The development is here equivalent to that of *Lufengosaurus*^15,16^. The coracoid tubercle is visible both in lateral and medial views, and is situated in the median area of the bone, near the dorsal margin. The posterior margin of the bone bears the posterodorsally oriented glenoid cavity. When in articulation, the angle between the coracoid component of the glenoid and the scapular one is of approximately 100°. The coracoid glenoid surface overhangs a posterolateral facet, which is anteriorly bordered by the coracoid tubercle. This facet is present in most early-diverging sauropodomorphs, with some variation regarding its orientation.

#### **Forelimb.**

##### ***Humerus***

Both humeri were recovered. The right humerus is subcomplete, the left one is incomplete and partially covered with plaster (Figs. 9 & S1; Table S4).

The humerus is hourglass-shaped and slender. The humeral head is rounded and bears a distinct bulge positioned in the middle of the proximal margin. The proximal half of the humerus exhibits a concave anterior surface and a slightly convex posterior surface. The deltopectoral crest is incomplete, but does not look like it was very prominent, conversely to what is observed in *Lufengosaurus*^15,16^. Its development was probably more similar to that of *Yizhousaurus*^9^ or *Yunnanosaurus*^4,19^. In *Yizhousaurus*^9^, the deltopectoral crest arises quite distally relatively to the proximal margin of the bone whereas in *Irisosaurus*, as in *Xixiposaurus*^7^ or *Yunnanosaurus*^4,19^, the point of origin of the deltopectoral crest is closer to the proximal margin. The deltopectoral crest extends on 50% of the total length of the humerus.

The humerus diaphysis is short relatively to the total length of the bone, therefore its medial and lateral margins are strongly concave dorsoventrally, like in *Jingshanosaurus*^5^, *Yizhousaurus*^9^ and *Yunnanosaurus*^4,19^. Conversely, in *Lamplughsaura*^3^ and *Xixiposaurus*^7^, the diaphysis appears proportionately longer, with less marked concavities. In lateral view, the anterior and posterior margins of the diaphysis are subparallel. The diaphysis transverse width represents 35% of the maximum proximal extension of the humerus (all the Chinese taxa for which the humerus measurements are available: *Jingshanosaurus*^5^, *Lufengosaurus*^15,16^, *Yizhousaurus*^9^ and *Yunnanosaurus*^4,19^, have values ranging between 24% and 36%) and 41% of the maximum distal one (values ranging from 34% to 45%). In cross-section the diaphysis is oval, its transverse width being superior to its anteroposterior thickness. In some genera, such as *Lufengosaurus*^15,16^ or *Xixiposaurus*^7^, the section is circular.

The transverse distal extension of the humerus equals 85% of the proximal one. The anterior surface of the distal humerus bears a cuboid fossa, and the posterior surface a shallow olecranon fossa. The condyles are visible, but poorly developed, as often in non-sauropodan sauropodomorphs. In distal view, they are subequal in size: the radial (medial) condyle is circular, while the ulnar (lateral) condyle is oval-shaped, with an oblique long-axis.

##### ***Ulna***

Both ulnae were recovered in articulation with radii. Ulnae and radii were separated during the preparation of the specimen. As for scapulocoracoids and humerii, the right ulna is the most complete and best preserved one (Figs. 9 & S1; Table S4).

For description and measurements, ulnae are positioned with the long axis of their proximal end oriented strictly anteroposteriorly. The radial fossa is therefore lateral and the anteromedial and anterolateral processes project, respectively, anteriorly and laterally (Fig. 9n).

The ulna is a robust bone which, in length, represents 68% of the length of the humerus. Overall, all the ulnae known from Chinese forms have a close morphology, with both ends extended with respect to the diaphysis and the main axis of the bone rotated of approximately 40°. The proximal articular surface of the ulna has a long oval anteromedial process and a shorter anterolateral process, as in all non-sauropodan sauropodomorphs. Between both processes is a shallow radial fossa. Another concavity, as deep as the radial fossa, is visible on the medial margin. Posteriorly, the articular surface of the ulna bears the olecranon. It is a blunt projection, showing the same degree of development than in *Lufengosaurus*^15,16^, *Yizhousaurus*^9^ or *Yunnanosaurus*^4,19^.

The diaphysis is the thinnest part of the bone. Relative to the ends, it seems stockier than in like *Lufengosaurus huenei*^15^, but more gracile than in *Jingshanosaurus*^5^. Both anterior and posterior margins of the diaphysis are dorsoventrally concave, while in *Yizhousaurus*^9^ the posterior margin appears rather straight. In anterior view, the medial margin is concave and the lateral one is straight. In cross-section, the diaphysis is circular.

The posterodistal corner of the ulna projects posterodorsally, and is more dorsally located than the anteriorly projected anterodistal corner. Above this corner, the anterodistal surface of the ulna is contacting the radius. The distal articular surface of the ulna is subrectangular in outline.

##### ***Radius***

The right radius is completely preserved, whereas the left one has both ends damaged (Figs. 9 & S1; Table S4). For description and measurements, radii are positioned following the ulna orientation, with the long axis of their proximal end oriented strictly anteroposteriorly (Fig. 9o).

The radius represents 60% of the humerus length. It is a straight and slender bone, with both ends more extended than the diaphysis. Its proximal end is oval-shaped, with the long axis almost twice as long as the perpendicular one. The proximal articular surface is flat, and its medial margin articulates in the radial fossa of the ulna.

The diaphysis is the thinnest part of the bone, both transversely and anteroposteriorly. It is straight and has an elliptical cross-section, the anteroposterior expansion being superior to the transverse width.

The distal end of the radius is slightly more extended than the proximal one, transversely and anteroposteriorly. As it is always observed in non-sauropodan sauropodomorphs, the posterodistal corner of the radius projects further than the anterodistal one. A part of the posterodistal surface contacts the ulna. The distal articular surface of the radius is flat and subtriangular in outline.

##### ***Manus***

Both hands were preserved in close articulation, and are subcomplete. As for other elements, the right side is the most complete. It preserves two carpals, five metacarpals and eight phalanges (Figs. 10 & S1; Table S4).

The carpus only includes two ossified carpals, in partial connection. Based on the interpretation made by Läng & Goussard^20^ (fig. 5B), and on a compilation of fossil data, we tentatively identify the larger element as the centrale and the second one as the second distal carpal. The centrale was not in articulation with the manus, but the shape of the bone leaves no doubt concerning its identification. It is a flattened and suboval element, which is roughly 56 mm long. It is similar in shape to the centrale of *Jingshanosaurus*^5^, *Lufengosaurus huenei*^15^ and *Yunnanosaurus huangi*^4^. The margins of the centrale are rugose, and both its distal and proximal surfaces are flat to slightly convex. The other carpal, angular and large, was glued to metacarpal IV. It is very rare to see a carpal, especially this size, in full contact with the proximal surface of metacarpal IV (Sereno^21^: fig. 10; Goussard^22^: fig. 12). Given its size (circa 80% of the centrale length) and shape, it would be most likely that it is the second distal carpal, which was articulated with metacarpal II in vivo. However, in some cases, a large carpal was found in situ near metacarpal IV^23^. In this case, and still following the work by Läng & Goussard^20^, it would rather be the fourth distal carpal. A carpal homologous in size and shape has been described in *Lufengosaurus huenei*^15^.

The hand morphology is comparable to that of the genus *Jingshanosaurus*^5^ in terms of proportion. Based on relative elongation of metacarpals II to V and width over length ratio of the phalanges, the hand of this specimen appears stouter than that of *Lufengosaurus* *huenei*^15^ or *Yunnanosaurus huangi*^4^. It is, however, less stout than the hand of *Yizhousaurus*^9^.

Metacarpal I is robust and quadrangular (Table S4), as in most non-sauropodan sauropodomorphs. Its proximal width over length ratio is 94% (right side) and 114% (left side), that is inferior to that of *Jingshanosaurus*^5^ (130%), and similar to those of *Lufengosaurus* *huenei*^15^ (114%) and *Yunnanosaurus huangi*^4^ (94%). The overall morphology of the bone is homogeneous among Chinese taxa, with proximodistally concave medial and lateral margins, a transversely concave distal margin, and a sigmoid proximal margin. The torsion between the bone proximal and distal ends is slightly marked. Distal condyles are large and dorsoventrally deep, with the lateral condyle projecting more anteriorly than the medial one. On the proximolateral margin of metacarpal I, a concavity allows the articulation with metacarpal II. The latter is not positioned in the alignment of metacarpal I, but slightly more anteriorly.

Metacarpal II is more elongated than metacarpal I (Table S4). Still, it appears rather stout compared to the slender metacarpal II of *Lufengosaurus* *huenei*^15^ or *Yunnanosaurus huangi*^4^. It is hourglass-shaped with strongly concave lateral and medial margins, conversely to the metacarpal II of *Lufengosaurus* *huenei*^15^ which margins are less concave. The proximal part of metacarpal II exhibits two flat articular surfaces: one, dorsomedial, articulates with metacarpal I. The other, ventrolateral, articulates with metacarpal III. The distal condyles are not as prominent as in metacarpal I and are dorsoventrally lower than the proximal end of the bone.

Metacarpal III is short and more gracile than metacarpal II, particularly because of a thinner diaphysis (Table S4). However, it exhibits a stouter morphology than in several other Chinese taxa, such as *Lufengosaurus*^15,16^ or *Yunnanosaurus*^4,19^. The medial and lateral surfaces of the bone are both proximodistally concave. The proximal end of metacarpal III is more extended transversely than the distal end, like in most non-sauropodan sauropodomorphs. The dorsomedial area of the bone bears the facet for metacarpal II, the ventrolateral surface that for metacarpal IV.

Metacarpal IV is shorter than metacarpal III (Table S4), with proportions quite similar to that of *Yizhousaurus*^9^, but dissimilar to the slenderer metacarpals IV of *Lufengosaurus*^15,16^ and *Yunnanosaurus*^4,19^. Medial and lateral margins are proximodistally concave, and the proximal end is markedly transversely wider than the distal one. The proximomedial area of the bone exhibits a flat surface where metacarpal III articulates. Metacarpal V is not in contact with an articular facet, but rather with the proximolateral corner of metacarpal IV.

Metacarpal V is the shortest and the most astonishing of the manus. It is practically as wide proximally as long (Table S4), curved and very stout. Among non-sauropodan sauropodomorphs, this morphology with strongly concave lateral and medial margins is widespread, but two shapes coexist: a subsymmetrical one like in *Yunnanosaurus huangi*^4^ and an asymmetrical one, with one side of the proximal articular surface longer than the other, like in *Yizhousaurus*^9^. In this specimen, the proximal half of metacarpal V is strongly asymmetrical, the proximomedial corner being much more anterior than the proximolateral one. For this reason, the proximal articular surface shows a 100° angle between the area articulating with the carpus and the surface in contact with metacarpal IV. It is the first Chinese taxon to present such feature.

On the right manus, 8 phalanges were preserved on the digits I to III. The phalangeal formula is the following: 2-3-(4)-?-?. Phalanx III-2 was glued to metacarpal III by mistake; the first phalanx of this digit is missing given that, in all non-sauropodan sauropodomorphs, the third digit has 3 non-ungual phalanges (see^16,5,9^).

The digit I is abnormally enlarged compared to the others, like in all non-sauropodan sauropodomorphs. Phalanx I.1 is robust and short, with a roughly equivalent length and width (Table S4). It is twisted to fit the asymmetrical shape of metacarpal I. Proximally, the phalanx has dorsal and ventral intercondylar processes, while distally, condyles are rather large, with marked collateral fossae and a ginglymus more extended ventrally than dorsally. The sharp and hooked ungual of digit I is the largest element of the manus.

Non-ungual phalanges are almost as long as wide on digit II, and longer than wide on digit III. Collateral fossae and intercondylar processes are observed on all non-ungual phalanges. The ungual of digit II represents roughly 60% of the ungual of digit I in length, but is also sharp and curved, while the ungual of digit III is less hooked than the two others.

#### **Pelvic Girdle.**

##### ***Ischium***

The distal ends of both ischia are preserved, the most complete one is the left (Fig. 11; Table S5). For the description, the ischia are oriented with the long axis horizontal.

The ischial shaft is subtriangular in cross-section, as in all non-sauropodan sauropodomorphs, with rounded edges making it more drop-shaped. The dorsal margin of the shaft is thick, whereas the ventral margin is laminar. Towards the distal end of the ischium, the cross-section goes from subtriangular to suboval, and stays dorsoventrally higher than transversely wide. The dorsal margin gets transversely wider distally, but the ventral margin stays sharp.

The distal end of the ischium is markedly expanded dorsoventrally with respect to the shaft, and its posteroventral corner is not projecting ventrally as in *Jingshanosaurus*^5^ and *Yunnanosaurus youngi*^18^. In dorsal view, the ischium lateral margin appears concave on its distalmost part. The ischial ends have a subcircular outline, like in *Xixiposaurus*^7^, whereas other Chinese genera, such as *Lufengosaurus*^15^ or *Yunnanosaurus*^4,18^ have a more suboval outline. When in articulation, the distal ischia are heart-shaped.

#### **Hindlimb.**

##### ***Pes***

Only one partial left ungual phalanx is preserved. Given the size and depth of the preserved proximal half, it is most likely that the ungual comes from digit II. The ungual phalanx is curved and transversely compressed, with prominent dorsal process and flexor tubercle. The beginning of the nutrient groove is visible on the lateral surface, but not on the medial surface. The proximal articular surface is strongly concave dorsoventrally and suboval in outline. It is subdivided into two subequal depressions by a median crest.

Comparison with non-sauropodan sauropodomorphs from the Early Jurassic of Asia

Among non-sauropodan sauropodomorphs, 9 genera and 12 species other than *Irisosaurus yimenensis* were described from Early Jurassic deposits of Yunnan. Of those, *Gyposaurus sinensis* was first considered as a juvenile synonym of *Lufengosaurus huenei* by Galton^24^, hypothesis that might be confirmed soon for the holotypic material^25^. *Gyposaurus sinensis* was also referred to the genus *Anchisaurus* by Dong^26^, and later regarded as valid and distinct from *Anchisaurus* in Galton & Upchurch^27^. Pending a complete reexamination and taxonomic study of the material referred to *Gyposaurus sinensis*, we here consider it a nomen dubium and do not include it in the following comparisons. We concur with Zhang et al.^6^ for the referral of the genus *Chuxiongosaurus* to *Jingshanosaurus*, and therefore also exclude it from the comparisons.

The main differences between *Irisosaurus* and *Jingshanosaurus*^5,6^ are that *Jingshanosaurus* has a more elongated maxilla premaxillary ramus, larger and more numerous neurovascular foramina on the maxilla lateral surface, a maxilla nasal process with a thin base (Fig. 4b), and ischia distal end projecting more ventrally in lateral view.

*Lufengosaurus huenei*^1,15^ has a subtriangular maxilla premaxillary ramus, a large antorbital fossa, a lateral ridge on the lateral surface of the maxilla (Fig. 4c), and a proximal end of the scapula more extended anteroposteriorly than the distal end. *Lufengosaurus magnus*^16^ scapula has a distinct angle between the scapular blade and acromion and a less projecting glenoid, a humerus with a subcircular diaphysis, and stockier hand metacarpals.

The main differences between *Irisosaurus* and *Xingxiulong*^17^ include the presence of a well-defined antorbital fossa on the maxilla in *Xingxiulong*, as well as cervical vertebrae with a more projecting anteroventral corner in lateral view, and a robust scapula with both ends expanded.

*Xixiposaurus*^7^ differs from *Irisosaurus* in having a maxilla premaxillary ramus longer than high, a well-defined antorbital fossa, numerous neurovascular foramina on the lateral surface of the maxilla (Fig. 4e), and teeth with denticles and smooth enamel.

*Yimenosaurus*^8^ has a maxilla with an elongate and subtriangular premaxillary ramus, no visible perinarial fossa, several neurovascular foramina under the nasal process, and a thin base of the nasal process (Fig. 4f), as well as denticles on the teeth.

The main differences between *Irisosaurus* and *Yizhousaurus*^9^ are that the latter has a large well-defined antorbital fossa, a reduced perinarial fossa, a premaxillary ramus of the maxilla longer than high, and numerous large neurovascular foramina on the lateral surface of the maxilla (Fig. 4g). *Yizhousaurus* has also teeth with smooth enamel and a robust hand.

*Yunnanosaurus huangi*^2,4^ differs from *Irisosaurus* in having a maxilla with a long premaxillary ramus, no neurovascular foramina and a ventrally extended antorbital fossa (Fig. 4h), as well as a more gracile hand and ischium distal end clearly longer dorsoventrally than wide transversely. *Yunnanosaurus robustus*^19^ has a maxilla with a clear antorbital fossa and numerous large neurovascular foramina (Fig. 4i), as well as teeth with denticles and a triangular proximal articular surface of ulna. *Yunnanosaurus youngi*^18^ has longest cervical vertebrae than *Irisosaurus* with epipophyses on the posterior postzygapophyses, dorsal centra with strongly anteroposteriorly concave ventral surface and a marked projection of the ischia posteroventral corner in lateral view.

One additional genus, *Pradhania*^3^, comes from the Jurassic of India. The main differences between *Irisosaurus* and *Pradhania* are that *Pradhania* has a dorsoventrally lower maxilla with five foramina and a prominent longitudinal ridge, teeth with denticles and more elongated cervical vertebrae.

**Supplementary Figure S1**


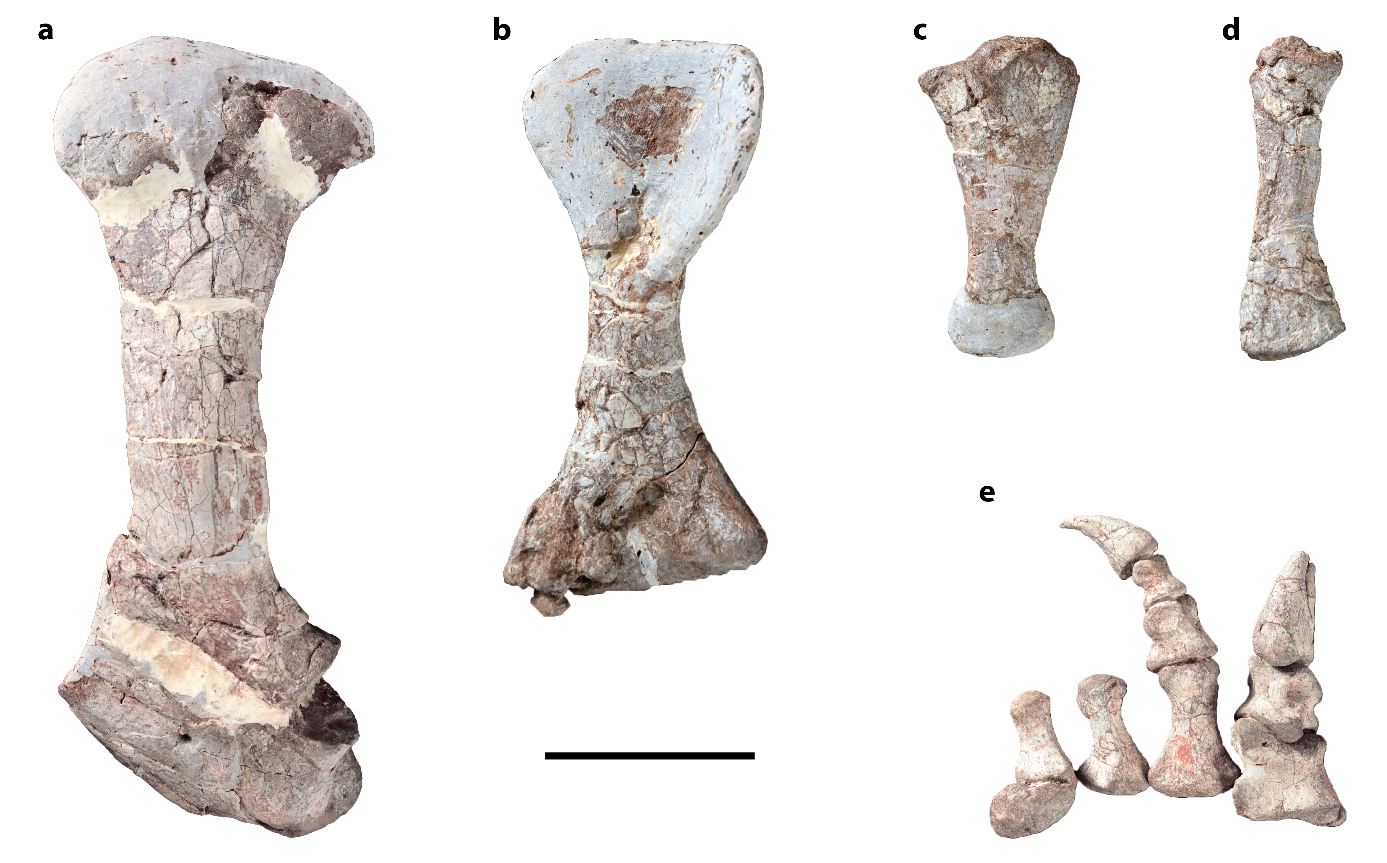
*Irisosaurus yimenensis* left scapular girdle and forelimb in lateral (**a**), anterior (**b-d**) and dorsal (**e**) views. (**a**) Scapulocoracoid; (**b**) Humerus; (**c**) Ulna; (**d**) Radius; (**e**) Manus. Scale bar = 10 cm.

**Supplementary Table S1**

Measurements (mm) of the vertebrae of *Irisosaurus yimenensis*. Abbreviations following the order of the table: **L**, maximum anteroposterior ventral length; **antW**, anterior width; **medW**, medial width; **postW**, posterior width; **antH**, anterior height; **postH**, posterior height; **naH**, neural arch height; **nsH**, maximum neural spine height; **nsL**, maximum neural spine length; **nsW**, maximum neural spine width measured at its distal end; **przD**, distance between the lateral margins of the prezygapophyses; **H**, total height of the vertebra; *****, deformation.

|  | CENTRUM | | | | | | NEURAL ARCH | | | | |  |
| --- | --- | --- | --- | --- | --- | --- | --- | --- | --- | --- | --- | --- |
|  | L | antW | medW | postW | antH | postH | naH | nsH | nsL | nsW | przD | H |
| Cant  C3-C5? | 98 | 51 | 28 | 63 | 57 | 57 | ? | ? | ? | ? | ? | >113 |
| Cmid  C5-C7? | 110 | 52 | 30 | 63 | 57 | 61 | ? | ? | ? | ? | 58 | >115 |
| Cmid  C6-C8? | 99 | 62 | 32 | 61 | 60 | 61 | ? | ? | ? | ? | ? | ? |
| Cpost  C9? | 97 | 63 | 32 | 73* | 62 | 60 | 90 | 40 | 41 | 32 | ? | 147 |
| Cpost  C10? | 85 | 67 | 37 | 77 | 64 | 70 | 70 | 64 | 42 | 46 | ? | ? |
| Dant  D2-6? | 75 | 60 | 24 | 66 | 65 | 64 | 60 | 50? | >60 | 10 | ? | 137 |
| Dmid  D5-9? | ? | ? | ? | ? | ? | ? | ? | 42 | 67 | 12 | ? | ? |
| Dmid  D5-9? | 79 | 51 | 32 | 59 | 55 | 59 | ? | ? | ? | ? | ? | ? |

**Supplementary Table S2**

Measurements (mm) of the scapulae of *Irisosaurus yimenensis*. Abbreviations following the order of the table: **L**, maximum dorsoventral length; **dW**, maximum distal width; **bW**, blade minimal anteroposterior width; **bT**, blade transversal thickness measured at midpoint; **pW**, maximum proximal width; **amxH**, acromion maximum height measured at the level of the point of divergence with the anterior margin of the blade; **amnH**, acromion minimum height measured on its distal end; **aW**, acromion anteroposterior width measured at the level of the anterior margin of the blade; **aT**, acromion transversal thickness; **gcT**, glenoid cavity maximum thickness.

|  |  |  | BLADE | |  | ACROMION | | | |  | |
| --- | --- | --- | --- | --- | --- | --- | --- | --- | --- | --- | --- |
|  | L | dW | bW | bT | pW | amxH | amnH | aW | aT | gcT | |
| Scapula (right) | 335 | 142 | 63 | 22 | 157 | 112 | 62 | 75 | 12 | 57 |  |
| Scapula (left) | 310 | ≈155 | 63 | 26 | ? | ? | ? | ? | ? | 60 |  |

**Supplementary Table S3**

Measurements (mm) of the coracoids of *Irisosaurus yimenensis*. Abbreviations: **L**, maximum anteroposterior length; **H**, maximum dorsoventral height; **H/L**, ratio height/length; **mW**, minimum transverse width measured on the anterior margin; **gcW**, glenoid cavity maximum transverse width.

|  | L | H | H/L | mW | gcW |
| --- | --- | --- | --- | --- | --- |
| Coracoid (right) | 165 | 120 | 73% | 11 | 50 |
| Coracoid (left) | ? | ? | ? | 9 | 55 |

**Supplementary Table S4**

Measurements (mm) of the forelimb of *Irisosaurus yimenensis*. Abbreviations following the table order: **L**, maximum proximodistal length; **dpcL**, deltopectoral crest proximodistal length (measured from the proximal margin of the humerus); **W**, maximum transverse width; **T**, maximum anteroposterior (dorsoventral for metacarpals and digits) thickness; **C**, circumference of the diaphysis (measured beneath the deltopectoral crest on humeri).

|  |  |  | PROXIMAL | | DIAPHYSIS | | | DISTAL | |
| --- | --- | --- | --- | --- | --- | --- | --- | --- | --- |
|  | L | dpcL | W | T | W | T | C | W | T |
| Humerus (right) | 245 | 126 | 132 | 45 | 48 | 38 | 145 | 117 | 46 |
| Humerus (left) | ≈250 | ? | ? | ? | 42 | 38 | 145 | 117 | 45 |
| Ulna (right) | 167 | - | 57 | 83 | 29 | 32 | - | 47 | 56 |
| Ulna (left) | 150 | - | 62 | 72 | 24 | 33 | - | ? | ? |
| Radius (right) | 146 | - | 31 | 47 | 21 | 26 | - | 40 | 48 |
| Radius (left) | 145 | - | 30 | 44 | 19 | 26 | - | 36 | 48 |
|  |  |  |  |  |  |  |  |  |  |
| Metacarpal I (right) | 52 | - | 49 | 35 | 38 | 19 | - | 42 | 26 |
| Metacarpal I (left) | 44 | - | 50 | 31 | 38 | 17 | - | 42 | 27 |
| Metacarpal II (right) | 60 | - | 42 | 22 | 19 | 16 | - | 27 | 18 |
| Metacarpal II (left) | 57 | - | 40 | 24 | 19 | 14 | - | 31 | 17 |
| Metacarpal III (right) | 59 | - | 32 | 21 | 14 | 13 | - | 23 | 20 |
| Metacarpal III (left) | 57 | - | 33 | 19 | 15 | 13 | - | 24 | 19 |
| Metacarpal IV (right) | 48 | - | 30 | 26 | 16 | 12 | - | 19 | 17 |
| Metacarpal IV (left) | 47 | - | 26 | 26 | 15 | 12 | - | 20 | 16 |
| Metacarpal V (right) | 34 | - | 31 | 23 | 20 | 14 | - | 25 | 19 |
| Phalanx I-1 (right) | 40 | - | 36 | 32 | - | - | - | 35 | 31 |
| Phalanx I-1 (left) | 39 | - | 38 | 33 | - | - | - | 34 | 27 |
| Phalanx I-2 (right) | 63 | - | 29 | 45 | - | - | - | - | - |
| Phalanx I-2 (left) | ? | - | 26 | 42 | - | - | - | - | - |
| Phalanx II-1 (right) | 30 | - | 29 | 27 | - | - | - | 25 | 18 |
| Phalanx II-1 (left) | 26 | - | 24 | 23 | - | - | - | 24 | 17 |
| Phalanx II-2 (right) | 23 | - | 23 | 19 | - | - | - | 19 | 13 |
| Phalanx II-2 (left) | 22 | - | 22 | 21 | - | - | - | 22 | 16 |
| Phalanx II-3 (right) | 39 | - | 17 | 25 | - | - | - | - | - |
| Phalanx II-3 (left) | ? | - | 19 | 26 | - | - | - | - | - |
| Phalanx III-1 (right) | 21 | - | 19 | 17 | - | - | - | 18 | 13 |
| Phalanx III-3 (right) | 16 | - | 13 | 14 | - | - | - | 13 | 11 |
| Phalanx III-4 (right) | 29 | - | 12 | 15 | - | - | - | - | - |

**Supplementary Table S5**

Measurements (mm) of the ischia of *Irisosaurus yimenensis*. Abbreviations following the table order: **L**, maximum proximodistal length; **W**, maximum (minimum for diaphysis) transverse width; **T**, maximum (minimum for diaphysis) dorsoventral thickness.

|  |  | PROXIMAL | | DIAPHYSIS | | | DISTAL | |
| --- | --- | --- | --- | --- | --- | --- | --- | --- |
|  | L | W | T | W | T |  | W | T |
| Ischium (right) | ? | ? | ? | ? | ? |  | 74 | 92 |
| Ischium (left) | ? | ? | ? | 37 | 46 |  | 65 | 90 |

**Supplementary Figure S2**


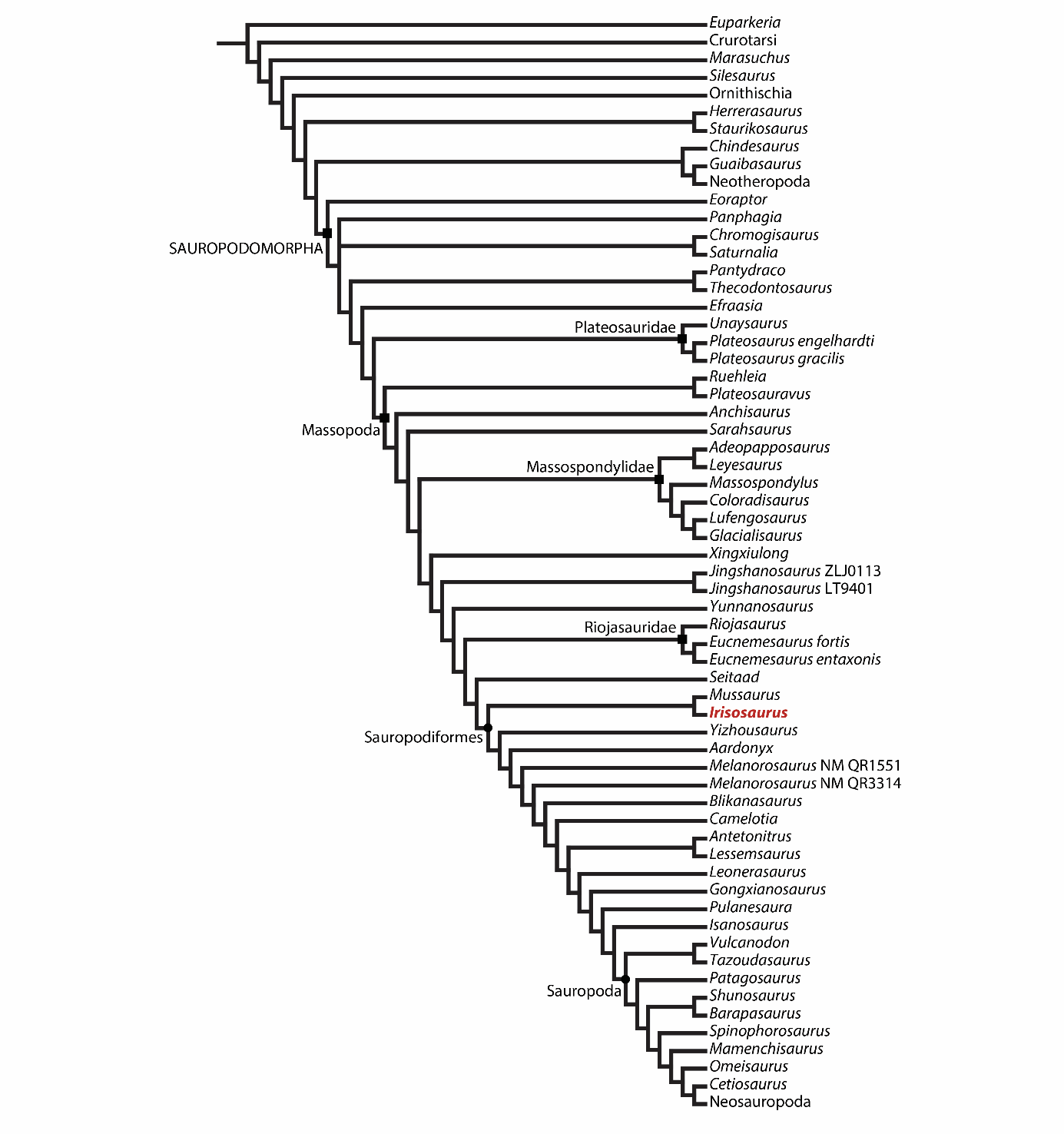
Strict consensus tree of the phylogenetic analysis conducted with 62 taxa and 364 characters, based on the matrix and scorings (except for *Irisosaurus*) of Zhang et al.^6^. Consensus tree of 2 MPTs with a length of 1300 steps (CI=0.33, RI=0.69). Squares represent stem-based definitions, ellipses represent node-based definitions.

**Supplementary Data S2**

Phylogenetic matrix

xread

364 62

Euparkeria 00000000?0000?0000000?000?100100000000000000000?00000000010010000000000000000?0000000?00?000?000000000?1000000000000000000?000000000000???0000??000000000?00?0?00??00000000000000000000000?00?0?????0????000?00200000000000??????00000000001?0?0?0000???00?000?0000?011000000000010000000000?02?000??0100000??1?000?100000?0000000??0?0?0010??0000?0????0?00?1?100000000?000

Crurotarsi 0000000000000?0[01]000000000?0001000000[01]0000000000000000000010000000000000000000?000000000000000000000000000000000000000000000000000000000???0000??000002000?00?0?00??0000200000000000000000000000000000[01]00?0[01]0?002?000000000000000000000000000000000000[01]00000000?0000?0[01]0000000000000000000000?00?0000000000000-1?0000[01]00000?0000000??0?0000000000?00000000?00?00100000000000[01]

Marasuchus 00???????0?0????????????0?00???????0???????????0???????????????????????????00???00000???????????????????????00?00000000?1?1000000000100???00?0??00?000000?00???00??000?00000000?000000000??0010100000000?000?012?1000?0000??????????????????????????????00000000000?0100010000000000000010000000110001000000??00000010001000100100000?0?0000000000?000??0?00?0?00?0??00???10

Silesaurus 00?0??00?0000?00100000??0?00?0?01010?????????0010??0????01?????????00?0?0?0000??000000?????1?00?0?010???0?0000010001000010?000000000000???0010000000000010100001100000000000000??100000100000?0?0????????111?11100000100000?????????????????????????????0000000000200100000000?010100?0?1000011011000100100000100011110000000?0000000??011?1??002???00000000?0?10???000?0011

Ornithischia 0010000000000?0000000?000?1011000100100?1000000100100001000000[01]000100100000000100000000010010[01]01[01]0000001001110011101000010000000000100100[01]0000??010000000?00?0?00?000000000000000[12]100000000000010000010000100111[02]110000010000000000000000000000000000000011012100010001??000?00110000000100001?0101111020000?000000[01]101110002?01?[01]1000000111100000?000?00?00?0[01]0000000000010

Herrerasaurus 00000000?0101000000000000-00000000000000100000010001010101000100001101000000101?0000000?1000?00000000?10000000000000000010100010000000100000?000010000011??0010[01]1000000200000010000001011001001100100100?1110??00110000000000000120000001000001101101001000002000000010000010??01?010100100001011100010110000010000110001000100100101000000011000001000000010001000000000?02

Staurikosaurus 00???????????????????????????????????????????????????????????????????????????????????????????0000000001000?00?0000000000????????00010?????00?01001?000011?000?00000000020000001??010?101100?001100100????1?1????????????????????????????????????????????00000200110000110?010?0010?00000100001??000?0101?000??000001100000001????01????????????????????????????????????????1

Chindesaurus ????????????????????????????????????????????????????????????????????????????????????????????????????????????????????????????????0???????????????01?0??0?1??0?1??????000??00?????000????1?0?????1000?0?????????????????????????????????????????????????????????0???001??????0?????????????00001011100010111000??????11111100???01?110100????????????????????????????????????1

Guaibasaurus ???????????????????????????????????????????????????????????????????????????????????????????????????????????????????????????????????????????????????0???01??011001000000??00000??0000000?00000?01?0??0[01]00??1??????110????1?0???????0????110??0?1?00??0?0?000?0[12]10021011001000000010?1011000000100100001011010??[01]00?0?11111100100?01101?0001011100000000000001?011000000000011

Neotheropoda 00[01]1[01]002[01]010201001100001[01]000000000000110000000010[01]100[01]01000001[01]0001[012]0100000010110000001110001000000001?000000000000000001011001100000010000[01]1000010200001010110010000001000000000[12]100001100100000010010000100010?1100000100010001200000010?0001001100[12]0101010300021110101000011110000110100001?01100010000001000[01]01111111100200101100000111110000??0?0000000?010000000000012

Eoraptor 0001?001?01010?0011100111010010010000110100000?1001001010??0110000[12]0011??11?1????????????????00000010???0?0?00?1000000001?????1100000000010?1011??000??01?00??100?000??00000011??10????10???001?00??0000?0100??10?10??00100??????20?00011?00010?00000[01]010010020002101??0??00?0??10?1011?10??0?011000010110001?00?1?0111010??1?0?00?????001????0?000?????0?0??0010000000??000

Panphagia ?????????????????????0?1???????????????????0??1????????1?1?0???????10?1?111??????????????????0?0?0?1010000?10?0101000000????????001000100111?01101?000?01???01?10000?000000000??0??0-00??0000?01000?0?0??0111???????????????????????????????????????????0??0?1[01]0021010?00?000??11??0?110???????????????????????001001010100???0100?010?0????????????????0???????[01]???????????

Chromogisaurus ??????????????????????????????????????????????????????????????????????????????????????????????????????????????????????????????????????????????????????????????????????????????????10000??10?0??1?????????????????????????0??????????????????????????????0????11002102?????????????????????0??????1???10110????[01]00000[12]010100010???????????????????????????????0??0???000???10

Saturnalia 10????????????????????????1??1????00?100?0??????????????????10?????1??00???0?0??0?0000??????????01000??????0000100000000????????00100010010??01101?00000100001100100000000000000001000010???0??1000???0??111011111100010102?????????????????????????????0000011[01]021020000000010110010010000001001[01]00010110000000000010101000100100101000000001000000000000010011000?000?0010

Pantydraco ?0???0????????????[01]00?0???1??1????000100100000010?100001?00????????00?000?1?10??00100011?000?00?01010??0000?1001010100001001??11001100101001101101?00???1????1????????????????????????0??1?0000100000100?????11?01?00???????????????????????????????????0010021100001?????????011??10110?00??????????1?1?0000??0000010?01?001??????????????????000011100000101?000000001001?

Thecodontosaurus ?0????????????????????????1??1??1000??????0?0?0?0???000????????????10?0?0?1010??011000???????00?11010??????1100101010000?1??????0001001010?1?01101?000001??00110000000000000000?10000?0101000?01000?0?00?010011201100010100110001201000110000101000001010000021000001???0?000?011??01010?[01]000111100001011000??[01]0000010101100??011010100?????1100?0?111??0??1??000??00?0????1

Efraasia 100?1001?010??1?111?112?1110?100???00100100000??0?10??01?0??1??????10?000?1?????0110100??????10010010??11?011001010100001???1?110011001001011011010000001??001?000000000000000001?00000100000?01100000?0?0100112012000111001101012010?11000001110100010000100310001010000100000110010110100001111000010110000000000011101?001?011010?0??01?111000001??0?0?01?1?001[01]000010002

Unaysaurus 100110011010??1?111??1??1010?1011000????????????0??00????001???000??0?10??0?10??110100??????010?10110?????01100101010000???0?1?????0??1?0??????????0??0?11?00?10000?0??00000?????????????0?0???010000?00?010011?11200001100??????1????110????211???0??0??????????????????????????????????????????????????????????????110110???011010?00????????0???1100??????0??0???0??????1

Plateosaurus_gracilis ?00??001?0102?110111?1??1110?101??00?10?1010?0??0?10001100??10??????????????????1?????????????0??0110?????11100101010?001?????1100??001???01101101?00?001?100110000000000000010?101000010000000010000?00????????11200?01100?1011?10101010000???10?????0?0010031100102001010001111001011??[01]0001111000010110?0??10000?1??0110??????????????????????0??????????????0?0????????[23]

Plateosaurus_engelhardti 1001100110102011[01]111112111100101100001001010101100110011000110010011011011011011110110001011110010110101111110010101000011001111001100100[01]0110110100020011100110000000000000010100100001[01]0000000100000000010011111[12]000011001101111010111001001010000010000100311001020010100011110010111110001111000010110001010000111101100100110101000010111000011100000010110011000010003

Plateosauravus ???????????????????????????????????????????????????????????????????????????????????????????????????????????????????????????????10???00100101?01101?00[12]0011?00110000000011000000??10???011??00??110??0??????0???[23]01110011100????????????1????????????????0010031100001??????????11??10110?100?11?1000010110001?1000001110110??????0??????????????????110???0?????0??????????3

Ruehleia ????????????????????????????????????????????????????????????????????????????????????????????????????????????????????????????0111001?00????01???101?0020011?00110010000011000010??1000001?0?00?0110???????010011101200011100?0100??01011101000???0??00???00100311000020??1100010110?10110?1010010100001021000??10?00?111011001?0?0010100????????????????????????????????????3

Adeopapposaurus 1001100210002111?11?11011011111010000101101000110[01]11011111001000102101[01]01100101101010101101000001011[01]10101011111110[01]100111001111002000100101101101100[01]001110011000000000000001001101000100000001000000000010010[12]21100010100110111101111101100201000001000[01]1003100000[02]011011001?110010110010001101000010[12]10101000000011101100100111101000011101000011110000010200011000011001

Leyesaurus ?001?00?1000211??11?????1010?11010000101101?00?1001111111?00100010[02]0011?????????01?10?110??1?00?10110??1010?1111010[01]0001?1???1110020001001111011????0????????????????????????????????????0000?001????000??????????????????????????????????????????????????????????????????100???????????????????????????????????????????????????????????????010??????????0?102000????0???0??

Massospondylus 100110021000211111111121111011001000010110100011001101111000100010210100110010110101010?1010010010110101010110[01]1010110011100?11100200010011110110100020011100110000000000000010111010001[01]000000000000000?01[01]011121201011100110111101112100101211000002000010031000[01]0001101100101100101110[01]0101101000010[12]101010[01]000001110110010011110100?01111100001111100?010200011000021002

Coloradisaurus ?00?1002??10??111111?1??1110??011000?10??0??10?1001101110000100100?101001101101?110101?????1?1001011010011?1000101010001?11?0011002000100101?01100?00?001?1001100000000000000101?????00?11000?011???0????010010121110011?????????????????1???????????????0?0?31100???011?110020110010110000001101000010211101110000011101100??011110?001??????00001111110101020001[01]000021002

Glacialisaurus ??????????????????????????????????????????????????????????????????????????????????????????????????????????????????????????????????????????????????????????????????????????????????????????????????????????????????????????????????????????????????????????????????????????????????????????0???????????????0011????????????????0???1010[01]0????1100?0111111?1010???????????????

Lufengosaurus 100???02???0211?1?11?11111111110100101011010000101110101100010?1????0110??0110??11010100?011010010????0111?11011010100011?00?11100200?110111?011010002001110011000000000000001001101?0011000000[01]1000000000110111212[01]1011100110111101113100101201000102000010031100002011011002011001011001010110100001021110111010001110110010011110?00001?11100[01]0111101010102000110000[12][01]004

Sarahsaurus 10?1000?10?1201??11?????1110111???0?010?0?1010010?110001[01]10010?0???001000111001?010101011??1?10?10010?????011001010100011?0011110?10001111111011?1100200111001?00?0000000000010?110??00110000000?000000000100101[02]12100011001101111011?110?1012110000020000100310[01]000200101100101100101110??101101000010010?010100000?1?01100100110101?0001111101[01]01?11200?0102000?[01]000?1100[12]

Xingxiulong ??????????????????????1???1?1111???0?10?0????011001100110000?000101?01?00100101?0??101???????0?????????001??000?????????11001111001000110?01?011010002001110011000000000?00001100110???1110000001?????00?0000????120?011110????1??????1101??????????????001003110000[12]0?1011002?11???0?100101011110000101101010?00000[01]10?110010011010?0?0?011?001101110000001?210011000021002

Riojasaurus ????????????????????????????????????????????????????????????????????????????????????????????????????????????????????????1?0?1111001100111101?011[01]1?001001110011000000000000001010101?00110000?0110000????????10321210011100?????????????????????????????00100311001020?1010001???????????1?010101010010111?0101000001110110010??????????????????????????????????????????????

Eucnemesaurus_fortis ???????????????????????????????????????????????????????????????????????????????????????????????????????????????????????????????????????????????????0???01??001100[01]10000??00?0???????????10000?01100??????????11???????????????????????????????????????????????????????110???0?????????????0110111010?1010110???000001110110????????????????????????????????????????????????[23]

Eucnemesaurus_entaxonis ???????????????????????????????????????????????????????????????????????????????????????????????????????????????????????????????????????????????????0?0?01??0????0???000??0000?00?1011?0?10?00?01????0???????????????????????????????????????????????????0?1??3?000100??1??0?0??11?010110?1?1?01110100101011010?0?????110111?1?0??0??????0??????11011112??001?[12]?0011000?????2

Yunnanosaurus 100?1002??00??10011001??101?111????00101110?00??0?11011111001000001?01?0?00010???????????????000?0010???0???00010?21000?1???111100[01]100110100?0110100000011000110000000001000000?0101?10110000?00?0???0??001?0??121200?111[01]0??01??0011?2101100201000[01]020?0010031000000011010001?110010111?[01]0101101000010110101?101000110011001?011010100001111001?0?1110?0?011???11?0000110?2

Seitaad ???????????????????????????????????????????????????????????????????????????????????????????????????????????????????????????????????????????????????00?[01]?1??00??00?001????0000???????????????????????????0010011??110001111011?11100111310?00?2110??0???0?????????????0?1000001?????????????????????????????????0?00?11101?0???01101010?00??11?00??11????0?010???11[01]0000010??

ZLJ0113_skull 1001??0210102111012?????111?11111000?10??0?????1?01??111?0000000???101100000101?210101??0??1?10011010?001101100101001001????????????????????????????????????????????????????????????????????????????????????????????????????????????????????????????????????????????????????????????????????????????????????????????????????????????????????????????????????????????????????

LT9401_skull ?00101021010211111211?11111111111000010110?0?1?11011011110001000??1?0110?000101?21010?0??0???100110100001?01100101001001????????????????????????????????????????????????????????????????????????????????????????????????????????????????????????????????????????????????????????????????????????????????????????????????????????????????????????????????????????????????????

Anchisaurus 10???00??0102?1??11?????111011?010?001010?10001100111101100?10??????01?001101?1??012110??????10?10?000?10000000101?11?0111?0011???100?1001111011?10000?010?001100?000??0000000??01000?0???000?00?10?0????11[01]0??2?1100101100??0??1101011100100201000002000[01]1013100000?111110001011?001010010101101000010[01]1110?01000001110110?1?01?010???010111?0?0011????0?01?1?0010000010011

Mussaurus ????????????????????????????????????????????????????????????????????????????????????????????????????????????????????????????????001000??????10110??0000?1??00100000000?00000010??1?1??0111?00001110?0????0110??101100011110?1?11?00101210110120100000200???0?3100010?011??1001011??10110?10101101000010111101?100000[12]1101110100110101010??????011011110000011200111000011003

Yizhousaurus 110110021010211111101111101111011000011100100011001111111100??00???10?100?00101?20?10?000001010110010??0000110[01]1010110011?101111001000110101?011010[01]000011100[12]10000000001000010?0100000110000?01????????0010011121100011110??????0010?21011011010001020000100311000000110110020110010110?111011110000101111011?????????????????????????????????????????????????????????????[34]

Aardonyx 1101?00210002?11?11?????1110?1011000??????1010??0?11010??10?1????????1?0????????????0??????1??0?0?1?0?????11?00101011001?1??????0010?0110100101101?0000011?00110001000020000010?1100??0110000?01110?0?00?????11??????????10??00??????13101???1?10??10????????????????111010001011??10110?101?1111000011110001????????1101110?0???01???????????01201111[02]0?0010?0?2??00?0???03

Leonerasaurus ?0????????????????????????????????????????????????????????????????????????????????????????????????0?0???????1?11010110011???001??010?[01]1???????1100?00?0?10000110000000??00000100?111?001?????????????????0100????1100000?????????????????????????????????11013100?????????0????11??1?1????0????????????????????????????????????????????????????100111020????????????0???????

Melanorosaurus_NMQR3314 1001?1031000211111101?1110101111???1010101101011001101011?00000010220110110010??2002010011011101?00111?1000110?10021100110?01011001?001101001011[01]10000001??0??100??0?0?10000000?01111001?0100?0?1??00?????1????[23]01100?0?110??????0010?2100101[12]0?000102000010031000[01]0[12]???????????1??10110?????????????1???1101110000011?011?01?0??0?0???100111001201111201001?2?011[12]000011003

Melanorosaurus_NMQR1551 ??????????????????????????????????????????????????????????????????????????????????????????????????????????????????????????????????????????????11???00?001??00210000000010000000?02?1100120000?0[01]11000????0100??2?????000110???????????21????????????????001003100000[01]011010001???????????11101111001110[01]11101110000011101100100?101010000??????11011112?000?????1120000????3

Blikanasaurus ???????????????????????????????????????????????????????????????????????????????????????????????????????????????????????????????????????????????????????????????????????????????????????????????????????????????????????????????????????????????????????????????????????????????????????????????????????????????0000?1110111?1?01001011111011100120111100111112102120000100?2

Camelotia ???????????????????????????????????????????????????????????????????????????????????????????????????????????????????????????????????1????????????0??0??001??002100000000??00?0???????????1???0??011????????????????????????????????????????????????????????????????????1?????????1??00111?111011110111?1??110???00000????????????????????????????????????????????[12]???000????4

Antetonitrus ????????????????????????????????????????????????????????????????????????????????????????????????????????????????????????????????0?[01]0????????????00?00?001??00[12]10002010121001000???1?????1??00?01100?0??0?0001??3211000[01]0110??????0010?3100???1010???????001003???0002010010001???????????[12]110111100111111110??1000002100101010?????????????????121111120100?????[12]1200?0????[34]

Lessemsaurus ????????????????????????????????????????????????????????????????????????????????????????????????????????????????????????????????0?011?1?0??0???101?00?0011100[12]1000[01]010111000000??????????????????????????0001???21?00011110???????0???310????1110??1????001003100000?011011001??1??10110?1?011111001011111?010?00000[01]1?0111???0110110001????????201111?????1?210[12]??00??????4

Gongxianosaurus 1?????0??????????12???????????????????????????????????????????????????????????????????????????????????????00???1??2121????????????0?????????????1??0??001??0??????0?0??00000????????????????0?0?1?0??000?011???2?110????1???????????????????????????????0?100???0????????????????????????[12]111?2??????0???110??1??????1?0??1???0?1?1?????1?11??01?0??????1?0??2?1112000020005

Pulanesaura ???????????????????????????????????????????????????????????????????????????????????????????????????????????????10?012011????????001?111?0100??11?????0??1??00[12][01]?0010?0120001000?????????2?111?011???0?0??????????????10??[12]?????????????????????????????????????????????????????11??1?11????????????????????????000002100101????????????????????????????????????????1????????

Isanosaurus ????????????????????????????????????????????????????????????????????????????????????????????????????????????????????????????????0??0????????????2??10??0???????????????2?011000?????????????????????????101?0????????????????????????????????????????????????????????????????????????????211112??0???001?1?011?????????????????????????1???????????????0???????????????????3

Tazoudasaurus 11??????????????????????????????????????????????????010??1?????????[02]011???1???1??????????????00?00000???0??10?1111012101???000??0010120??????0002??100001??00200101011020121000?????????21?11?111?????11?????01?22100100?[12]????????010?[01]10000100?0001?310?1???3101??0?111000002?11??1??1??21101111001111011?0???0010020101010?1101011111??011????1?????20????????1??10?1????5

Vulcanodon ?????????????????????????????????????????????????????????????????????????????????????????????????????????????????????????????????????????????????????????????[12]????????????????????1??????01?11?0?00??0?0???1???2?[12]10?10?0[12]1??????????????????????????????????310[12]?00?0111100010111110010?111011110010011?1????[12]0?10021?0???11?10101101111011??111?10102?1001020111110?121?05

Patagosaurus 11????????????????????????????????????????????????????????????????????????????????????????????1?00001??????0??0111012111??????11001012100000110020?2001010?00201?02111120121?00?02100011[12]0?01?0?100????0?111?00??2100100?21?????????????????????????????11110310100001111100110111110010?211112??0???0011111???10100[01]??0???????????????????????????????????????????????????5

Barapasaurus ???????????????????????????????????????????????????????????????????????????????????????????????????????????????1?111211???????????1?1?????00??002??100101??00201102111120121100?0[12]10001?1?1?1??1????0????101?00?21???????21?????????????????????????????1111031011000111110011??11110010???????????????????1???1?1?????????1?1???????1??????????20????????????????2?1??????5

Shunosaurus 11000113?110201101200000001011?20100111201010101001101012000010011121010001011?120020???110200110000120100011111112121112??00011?10012?1?10010012?1100001011021?0??01??201210000?11??00?101?110110011?111?1?0002221001100?11000?1011001?000110000??1?310111103101100011111?0110?111100100211112??0???000?011??21010?[01]1?0????110?101???1?1011??1121?110??2?00?2?12121?1021105

Spinophorosaurus 1????1?????????????????????????????????2????????????0????01?????????0??101111110[12]00???????????1?????????????100111012?112?????11?110101?0?01?0002?110??01????[12]?11???1?12?1010?0?????????[12]???11011?001?1??1110?03?2[12]0?10?????????????????????????????????????????[12]????1?1???01[12]?011??001??211111110?1101??0?0??20?1002??0??11?10??0?0?01?????????????????????????????????????

Mamenchisaurus 11000113?11120110120000000101112??001112010101011111110121100000111221010000111?201?00?????2?11[01]0000100100011111111121113?00011101210010000110002?1110101010020110?011020121000001100111211111011001?0111111000322100100120110011011000100011?0?10?1?31?1111031011?0011111001100110100100211112??0???010?011??21010?0??0??????1010111111??????1120?1????2?1??2?121211102??05

Omeisaurus 110001131111201101200000101011?201001112010101011111010121100000112221110010111???0?00???1???11100001?0?00011101111121113??0011101200011010111002?1210101011020110?011020121100?021???1?20111100100??0111101000322100100021??????0100001000110001001?310111103101100011111001100111100100211012??0???010?010??210100?1?0??111?011010?11???1????121?110??2?10?20121211102?105

Cetiosaurus 1????????????????????????????????????????????????????????11?????????????????????????????????????????????????????????????2??00111101110110100100020?2001?1??1020??1[12]11102010100???[12]??????20111?00100?0??01111010321100100021???????1?????????????????????111103?0210?0110?1?011?1101?00???211112??0???010?111??210100210010111??????????????????????????????????????????????5

Neosauropoda 1100011311112011012000001010111[02]11101112010101011[01]11110121100[01]10112221110010111120[01]200000102011100001201000[01]111111[02]12[01]112[01]00001110011?110100110020121010101[01]020111[12]0110201211000021000112011111[01]1000[01]01001110003?21001[01]00211100010100000000110001??1?300111103102100011111001101110[01]10[01]00211012??0???010?1111121010?010000111110101111111011??11211100??2?10?201212111021105

Irisosaurus ?????????011??1???1?????10????1??????????????????????????????????????????????????????????????????????????????0?10-21100-????????000???????????110??00?0010??0??00000???0?0?00????????????????????????????010010?0?1??01?110??????1010121011012010000020?????????????????????????1??10110????????????????????????????????????????????????????????????????????????????0?0?????

;

ccode - 0.6 8.11 13.17 19.21 23.38 40.55 57.67 69.90 92.100 102.115 117.119 121.129 131 132 134.143 145 146 148 151.156 158.161 163.166 168 169 171.176 178.183 185.206 208 209 211.216 218.224 226.229 231.236 238.244 246.252 254 255 257.268 270.280 282.301 303.307 309.315 317.335 337.348 350 351 353 355.358 360.363 *;

proc /;

comments 0;

**Supplementary Data S3**

3D model of the maxilla of *Irisosaurus.*

See file “Irisosaurus Maxilla CEVB21901”

**References**

1. Barrett, P. M., Upchurch, P. & Wang, X.-L. Cranial osteology of *Lufengosaurus huenei* Young (Dinosauria: Prosauropoda) from the Lower Jurassic of Yunnan, People’s Republic of China. *J. Vertebr. Paleontol*. **25**, 806–822, 10.1671/0272-4634(2005)025[0806:COOLHY]2.0.CO;2 (2005).
2. Barrett, P. M., Upchurch, P., Zhou, X.-D. & Wang, X.-L. The skull of *Yunnanosaurus huangi* Young, 1942 (Dinosauria: Prosauropoda) from the Lower Lufeng Formation (Lower Jurassic) of Yunnan, China. *Zool. J. Linn. Soc*. **150**, 319–341, 10.1111/j.1096-3642.2007.00290.x (2007).
3. Kutty, T. S., Chatterjee, S., Galton, P. M. & Upchurch, P. Basal sauropodomorphs (Dinosauria: Saurischia) from the Lower Jurassic of India: their anatomy and relationships. *J. Paleontol*. **81**, 1218–1240, 10.1666/04-074.1 (2007).
4. Young, C. C. *Yunnanosaurus huangi* Young (gen. et sp. nov.), a new Prosauropoda from the Red Beds at Lufeng, Yunnan. *Bulletin of the Geological Society of China.* **22**, 63–104 (1942).
5. Zhang, Y. & Yang, Z. *A new complete osteology of Prosauropoda in Lufeng Basin, Yunnan, China*. (Yunnan Publishing House of Science and Technology, 1995).
6. Zhang, Q. N., Wang, T., Yang, Z. W. & You, H. L. Redescription of the Cranium of *Jingshanosaurus xinwaensis* (Dinosauria: Sauropodomorpha) from the Lower Jurassic Lufeng Formation of Yunnan Province, China. *Anat. Rec*. 10.1002/ar.24113 (2019).
7. Sekiya, T. A new prosauropod dinosaur from the Early Jurassic Lower Lufeng Formation in Lufeng, Yunnan. *Global Geology*. **29**, 6–15 (2010).
8. Bai, Z., Yang, J. & Wang, G. *Yimenosaurus*, a new genus of Prosauropoda from Yimen County, Yunnan Province. *Yuxiwenbo (Yuxi Culture and Scholarship)*. **1**, 14–23 (1990).
9. Zhang, Q. N., You, H. L., Wang, T. & Chatterjee, S. A new sauropodiform dinosaur with a ‘sauropodan’ skull from the Lower Jurassic Lufeng Formation of Yunnan Province, China. *Sci. Rep.* **8**, 13464, 10.1038/s41598-018-31874-9 (2018).
10. Hendrickx, C., Mateus, O., & Araújo, R. A proposed terminology of theropod teeth (Dinosauria, Saurischia). *J. Vertebr. Paleontol*. **35**, e982797, 10.1080/02724634.2015.982797 (2015).
11. Pol, D., Garrido, A. & Cerda, I. A. A new sauropodomorph dinosaur from the Early Jurassic of Patagonia and the origin and evolution of the sauropod-type sacrum. *PLoS ONE*. **6**, e14572; 10.1371/journal.pone.0014572 (2011).
12. McPhee, B. W., Bonnan, M. F., Yates, A. M., Neveling, J. & Choiniere, J. N. A new basal sauropod from the pre-Toarcian Jurassic of South Africa: evidence of niche-partitioning at the sauropodomorph–sauropod boundary? *Sci. Rep*. **5**, 13224, 10.1038/srep13224 (2015).
13. Upchurch, P. The phylogenetic relationships of sauropod dinosaurs. *Zool. J. Linn. Soc*. **124**, 43–103 (1998).
14. Chure, D., Britt, B. B., Whitlock, J. A., Wilson, J. A. First complete sauropod dinosaur skull from the Cretaceous of the Americas and the evolution of sauropod dentition. *Sci. Nat*. **97**, 379–391, 10.1007/s00114-010-0650-6 (2010).
15. Young, C. C. A complete osteology of *Lufengosaurus huenei* Young (gen. et sp. nov.) from Lufeng, Yunnan, China. *Palaeontologia Sinica C*. **7**, 1–53 (1941).
16. Young, C. C. On *Lufengosaurus magnus* Young (sp. nov.) and additional finds of *Lufengosaurus huenei* Young. *Palaeontologia Sinica C*. **12**, 1–53 (1947).
17. Wang, Y. M., You, H. L. & Wang, T. A new basal sauropodiform dinosaur from the Lower Jurassic of Yunnan Province, China. *Sci. Rep.* **7**, 41881, 10.1038/srep41881 (2017).
18. Lü, J. *et al*. New yunnanosaurid dinosaur (Dinosauria, Prosauropoda) from the Middle Jurassic Zhanghe Formation of Yuanmou, Yunnan Province of China. *Memoir of the Fukui Prefectural Dinosaur Museum*. **6**, 1–15 (2007).
19. Young, C. C. The Lufeng saurischian fauna in China. *Palaeontologia Sinica C*. **13**, 1–96 (1951).
20. Läng, É. & Goussard, F. Redescription of the wrist and manus of ?*Bothriospondylus madagascariensis*: new data on carpus morphology in Sauropoda. *Geodiversitas*. **29**, 549–560 (2007).
21. Sereno, P. C. Basal Sauropodomorpha: historical and recent phylogenetic hypothesis, with comments on *Ammosaurus major* (Marsh, 1889). *Special Papers in Palaeontology*. **77**, 261–289 (2007).
22. Goussard, F. *Étude morpho-fonctionnelle de la main des dinosaures sauropodomorphes : implications évolutives et apport de l’analyse en éléments finis*. (PhD Thesis, Muséum National d’Histoire Naturelle, Paris, France, 2009).
23. Sertich, J. J. W. & Loewen, M. A. A New Basal Sauropodomorph Dinosaur from the Lower Jurassic Navajo Sandstone of Southern Utah. *PLoS ONE*. **5**, e9789, 10.1371/journal.pone.0009789 (2010).
24. Galton, P. M. Prosauropod dinosaurs (Reptilia: Saurischia) of North America. *Postilla*. **169**, 1–98 (1976).
25. Wang, Y., You, H., Otero, A. & Wang, T. Taxonomy of *“Gyposaurus” sinensis* Young, 1941 from the Early Jurassic Lufeng Formation of Yunnan Province, Southwestern China in *Society of Vertebrate Paleontology, Meeting Program & Abstracts*: 210 (2017).
26. Dong, Z. 1992. *Dinosaurian faunas of China*. (China Ocean Press, 1992).
27. Galton, P. M. & Upchurch, P. Prosauropoda in *The Dinosauria, Second Edition* (eds Weishampel, D. B., Dodson, P. & Osmólska, H.) 232–258 (University of California Press, 2004).
